# Quantitative Attribution of the Protective Effects of Aminosterols against Protein Aggregates to Their Chemical Structures and Ability to Modulate Biological Membranes

**DOI:** 10.1021/acs.jmedchem.3c00182

**Published:** 2023-07-11

**Authors:** Silvia Errico, Giacomo Lucchesi, Davide Odino, Enass Youssef Osman, Roberta Cascella, Lorenzo Neri, Claudia Capitini, Martino Calamai, Francesco Bemporad, Cristina Cecchi, William A. Kinney, Denise Barbut, Annalisa Relini, Claudio Canale, Gabriella Caminati, Ryan Limbocker, Michele Vendruscolo, Michael Zasloff, Fabrizio Chiti

**Affiliations:** † Department of Experimental and Clinical Biomedical Sciences, Section of Biochemistry, University of Florence, Florence 50134, Italy; ‡ Centre for Misfolding Diseases, Department of Chemistry, University of Cambridge, Cambridge CB2 1EW, UK; § Department of Chemistry “Ugo Schiff” and CSGI, University of Florence, Sesto Fiorentino 50019, Italy; ∥ Department of Physics, University of Genoa, Genoa 16146, Italy; ⊥ Department of Pharmacology and Toxicology, Faculty of Pharmacy, Tanta University, Tanta 31527, The Arab Republic of Egypt; # European Laboratory for Non-linear Spectroscopy (LENS), Sesto Fiorentino 50019, Italy; ∇ Department of Physics and Astronomy, University of Florence, Sesto Fiorentino 50019, Italy; ○ National Institute of Optics, National Research Council of Italy (CNR), Florence 50125, Italy; ◆ Enterin Research Institute Inc., Philadelphia, Pennsylvania 19103, United States; ¶ Department of Chemistry and Life Science, United States Military Academy, West Point, New York 10996, United States; & MedStar-Georgetown Transplant Institute, Georgetown University School of Medicine, Washington, District of Columbia 20007, United States

## Abstract

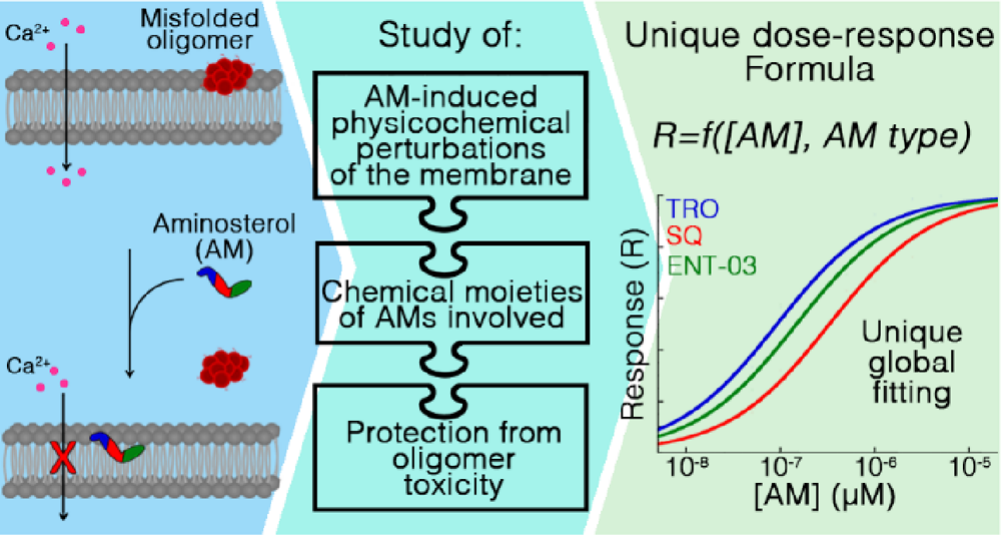

Natural aminosterols are promising drug candidates against neurodegenerative diseases, like Alzheimer and Parkinson, and one relevant protective mechanism occurs via their binding to biological membranes and displacement or binding inhibition of amyloidogenic proteins and their cytotoxic oligomers. We compared three chemically different aminosterols, finding that they exhibited different (i) binding affinities, (ii) charge neutralizations, (iii) mechanical reinforcements, and (iv) key lipid redistributions within membranes of reconstituted liposomes. They also had different potencies (EC_50_) in protecting cultured cell membranes against amyloid-β oligomers. A global fitting analysis led to an analytical equation describing quantitatively the protective effects of aminosterols as a function of their concentration and relevant membrane effects. The analysis correlates aminosterol-mediated protection with well-defined chemical moieties, including the polyamine group inducing a partial membrane-neutralizing effect (79 ± 7%) and the cholestane-like tail causing lipid redistribution and bilayer mechanical resistance (21 ± 7%), linking quantitatively their chemistry to their protective effects on biological membranes.

## Introduction

Many of the most severe neurodegenerative diseases originate from the conversion of specific polypeptide chains from their native soluble states into amyloid aggregates, forming various types of extracellular deposits or intracellular filaments, including amyloid plaques by the amyloid-β (Aβ) peptide, neurofibrillary tangles by the tau protein, Lewy bodies by α-synuclein (αS), amyloid plaques by the prion protein (PrP^sc^), and numerous others.^1,2^ Their corresponding disorders are Alzheimer’s disease (AD), frontotemporal dementia (FTD), Parkinson’s disease (PD), spongiform encephalopathies (SE), and many other pathologies.^1,2^ Key pathogenic species in these disorders are thought to be small oligomeric species. These transient aggregates form both during the process of amyloid fibril formation and upon their release from mature fibrils, and oligomers are able to interact with a number of molecular targets, including biological membranes, giving rise to a cascade of dysfunctional events.^1,3^

Aminosterols (AMs) isolated from the dogfish shark *Squalus acanthias* have been increasingly shown to change amyloid fibril formation for Aβ and αS dramatically^4−6^ and inhibit the interaction of amyloidogenic proteins with biological membranes, both in their monomeric and oligomeric forms,^4−9^ thus representing putative drug candidates against AD, PD, and possibly other neurodegenerative conditions.^10^ In particular, two of them have been shown to decrease significantly, or even eliminate, the toxicity of misfolded αS or Aβ oligomers in cell cultures and *C. elegans* models of PD and AD by interacting with cell membranes and either preventing the binding or displacing oligomers from the plasma membrane.^4−8^ AMs have also been shown to bind to the lipid bilayer of membrane liposomes^9,11^ and exert their monomer/oligomer displacement or binding inhibition effect even on such reconstituted systems devoid of proteins,^4−6,9^ suggesting that they mediate their effect through membrane bilayer binding. AMs were not found to bind to oligomers in a way that alters their morphology and structure at concentrations where they exhibit membrane binding and cell protection,^7,8,10^ ruling out that oligomer-AM binding is responsible for the AM-mediated inhibition of oligomer-membrane binding.

**Figure 1:**
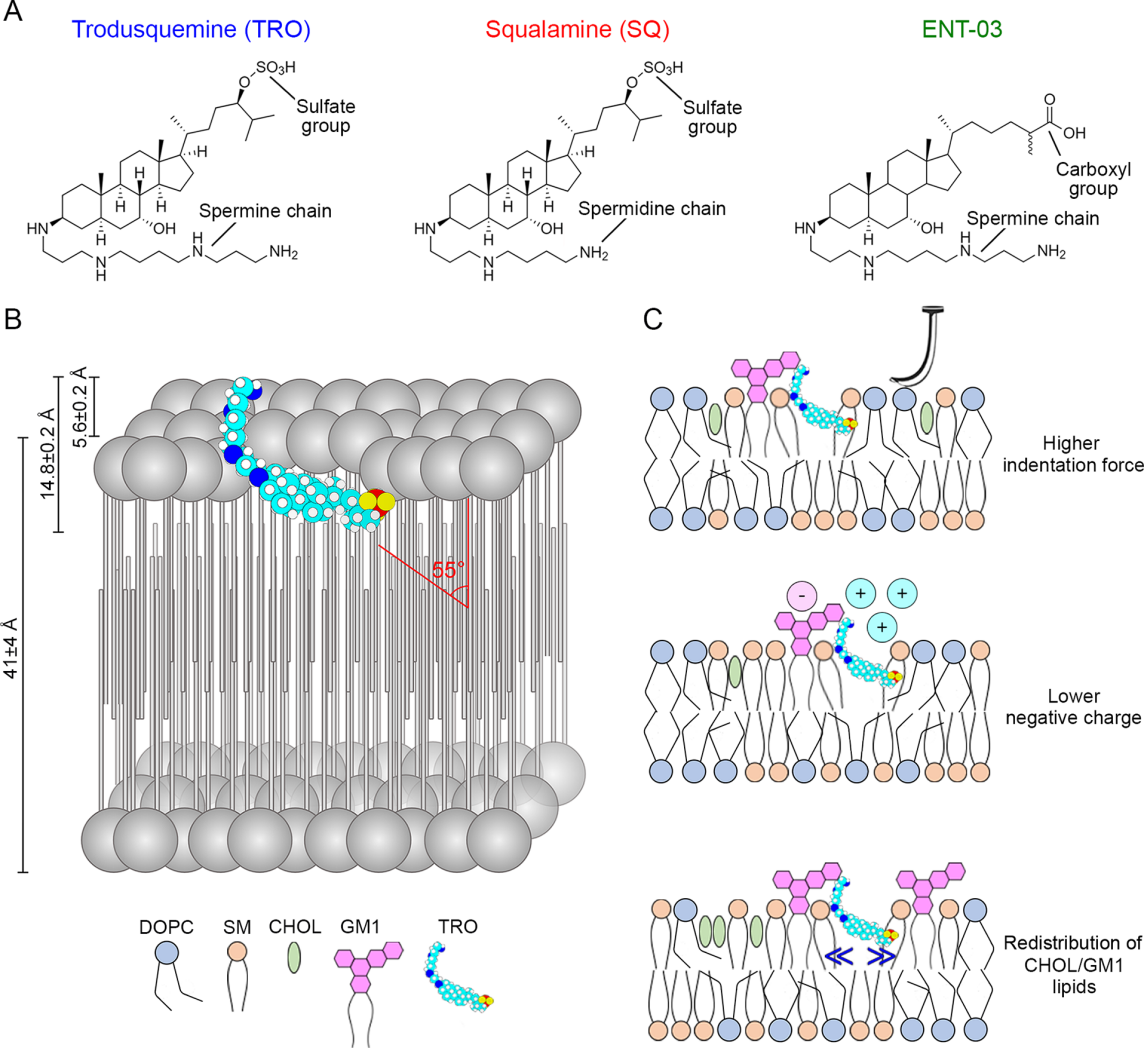
Structural formulas of the three AMs investigated in this work, and mode of membrane insertion and perturbation for TRO. (A) Chemical structures of TRO (blue), SQ (red), and ENT-03 (green). (B) Schematic representation of the insertion and localization of TRO within biological membranes, as previously determined experimentally.^11^ The 55° angle refers to the whole molecule, rather than the steroid group or polyamine group only. The 14.8 ± 0.2 and 5.6 ± 0.2 Å distances refer to the space occupied by the molecule and portion sticking out of the membrane along the normal to the bilayer plane, respectively. (C) Schematic representation of the three major physico-chemical effects on cell membranes induced by the insertion of TRO, all possibly mediating the TRO-induced protection against the toxicity of misfolded protein oligomers.^11^

These compounds are known as squalamine (SQ) and trodusquemine (TRO), which are also known as ENT-01 and MSI-1436, respectively (Figure 1A). In mouse models of PD and in wild-type aged mice, SQ has been found to restore excitability within the enteric neurons and to restore normal colonic motility.^12,13^ SQ (in the form of its phosphate salt, ENT-01) has just completed a multicenter, randomized, double-blind, placebo-controlled phase-2b clinical trial in patients with PD-related constipation (KARMET, identifier NCT03781791) and shown improvement in constipation as well as hallucinations and dementia.^14^ A previous multicenter, open label phase 2 study in patients with Parkinson’s disease (RASMET, identifier NCT03938922) had also shown improvement in constipation, sleep, REM-behavior disorder, hallucinations, and dementia.^15^ Both TRO and SQ have passed phase 1 clinical trials showing good safety and tolerability (Identifiers: NCT00606112 and NCT00139282, respectively).

The interaction of AMs with the lipid bilayer of biological membranes is, therefore, a central component in the mechanism by which these small molecules mediate their protection against amyloidogenic proteins and their misfolded oligomers. This interaction has been mainly investigated, at the physicochemical and molecular levels, using TRO and liposomes in the form of large unilamellar vesicles (LUVs) as a model of the lipid bilayer of the cell membrane.^10,11^ TRO was reported to stably insert into the hydrophilic portion of the first upper layer of the membrane, down to the interface between the hydrophilic and hydrophobic portions, exposing both sulfate and spermine groups to the aqueous phase (Figure 1B).^11^ The molecule digs into the membrane by 9.2 ± 0.2 Å, with an angle of 55°, and has its spermine moiety sticking out of the membrane by 5.6 ± 0.2 Å, occupying a total length of 14.8 ± 0.2 Å along the axis perpendicular to the bilayer plane (Figure 1B). The insertion of TRO in lipid bilayers leads to three main physicochemical perturbations of the membrane (Figure 1C): (i) a decrease of the total negative charge, (ii) an increase of the mechanical resistance to indentation, and (iii) a reorganization of the spatial distribution of both cholesterol (CHOL) and monosialotetrahexosylganglioside 1 (GM1) lipids, clustering CHOL molecules, clustering GM1 molecules, and separating CHOL from GM1.^11^ These three known perturbations have been postulated to reinforce biological membranes against the toxic action of protein misfolded oligomers.

In spite of these recent advances, it is not yet clear which of the three aforementioned changes of the membrane bilayer induced by AMs are the most effective in mediating their protective role and if all of them are relevant. Moreover, it remains to be elucidated which of the chemical moieties of the AMs are mainly involved in mediating their protection. These drawbacks limit our ability to predict the potency of other natural AMs and to anticipate the potency of other related or newly designed molecules. More generally, they limit our understanding of the relationship between the chemistry of small molecules, the physicochemical status of the cell membrane, and the toxicity of misfolded oligomers, thereby hindering the search of generic strategies to protect cell membranes from the action of deleterious protein oligomers.

In this work, we compare three different chemically synthetized AMs, characterized by different chemical and structural formulas. We compare, in particular, SQ, TRO, and ENT-03, the latter of which is an AM recently identified in the mouse *Mus musculus*,^16^ whereas TRO and SQ were originally isolated from the dogfish shark *Squalus acanthias*.^17,18^ The three AMs share a sterol group, an alkyl moiety of the cholestane-type fused to the sterol at C-17, and an alkyl polyamine tail fused to the sterol at C-3 and replacing the hydroxyl group (Figure 1A), as previously described.^16−18^ However, the three AMs differ because of a sulfate moiety at position 24 for SQ and TRO, and a carboxylate group replacing the methyl group at position C-25 for ENT-03 (Figure 1A). They also differ for the alkyl polyamine group, which is a spermidine for SQ (7 methylene and 3 amino groups) and a spermine for TRO and ENT-03 (10 methylene and 4 amino groups), resulting in a higher net positive charge by one unit relative to SQ (Figure 1A). Herein, we demonstrate that the three AMs bind to cell membranes with different affinities, affect the physicochemical and molecular properties of the lipid bilayer to different extents, and result in different protective effects, raising an opportunity to compare these different aspects at the chemical and physical levels. We then relate all these chemical, physical, molecular, and biological measurables using an approach of quantitative chemical biology that led to the precise identification and quantification of: (i) the chemical groups within AMs mainly responsible for their protective effect, and (ii) the specific physicochemical changes of the membrane that most mediate this protective effect, both enriched with a predictive power.

## Results and Discussion

### All Three AMs Bind to LUVs

To investigate whether TRO, SQ, and ENT-03 bind to LUVs, the fluorescence anisotropy (*r*) values of 10 μM BODIPY TMR-X-labeled AM (AM-BODIPY) and Alexa Fluor 594-labeled AM (AM-A594) were measured in the absence and presence of 0.5 mg/mL LUVs incubated with the three AMs for 15 min. BODIPY and A594 labeled the distal primary amino group of the polyamine chain, which is known to stick out of the membrane from previous studies on TRO^11^ and is not expected, therefore, to affect the AM-membrane binding affinity significantly. In addition, the two probes have different chemical properties. BODIPY has a lower molecular weight, is hydrophobic, and has a neutral net charge, whereas A594 has a higher molecular weight, is hydrophilic, and has a negative net charge. These differences enable the assessment of whether the probe chemistry affects dramatically the LUV-AM binding affinity.

**Figure 2:**
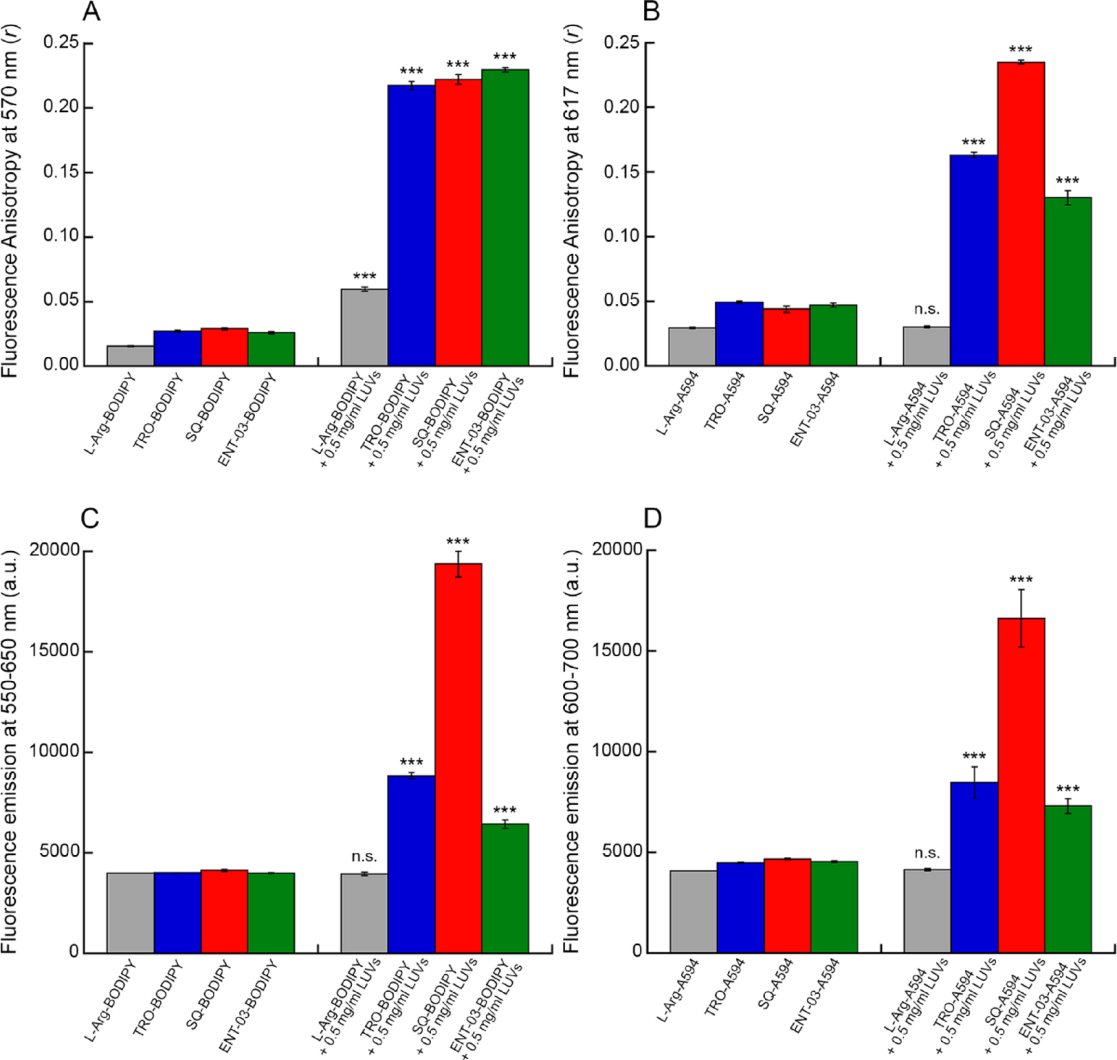
Changes in fluorescence anisotropy and emission of the three fluorescently labeled AMs in the presence of LUVs. (A, B) Fluorescence anisotropy (*r*) values at 570 nm for BODIPY (A) and at 617 nm for A594 (B), of 10 μM L-Arg (gray), TRO (blue), SQ (red), and ENT-03 (green) labeled with BODIPY (A) and A594 (B), obtained in the absence and presence of 0.5 mg/mL LUVs. (C, D) Fluorescence emission corresponding to the integrated area between 550–650 nm for BODIPY (C) and 600–700 nm for A594 (D), of 10 μM L-Arg (gray), TRO (blue), SQ (red), and ENT-03 (green) labeled with BODIPY (C) and A594 (D), obtained in the absence and presence of 0.5 mg/mL LUVs. Bars indicate mean ± SEM (*n* = 5. n.s., nonsignificant; ***, *p* < 0.001 relative to corresponding values in the absence of LUVs (Student’s *t*-test).

The incubation of all BODIPY-labeled AMs with LUVs showed a highly significant increment of *r* (*p* < 0.001, unpaired, two-tailed Student’s *t*-test, Figure 2A, Table 1), suggesting a lower rotational freedom of the probe in the presence of LUVs. A similar result was obtained with A594-labeled AMs (*p* < 0.001, Figure 2B, Table 1). *r* values of 10 μM L-Arg-BODIPY and L-Arg-A594, used here as negative controls of similarly labeled small molecules that have no predicted ability to bind to LUV bilayers, increased only marginally or did not increase significantly in the presence of LUVs (Figure 2A,B). L-Arg-BODIPY displayed a slight, but significant, increase of *r* when incubated with LUVs that was not reproduced with L-Arg-A594, possibly due to the hydrophobic nature of the BODIPY dye that caused clustering of L-Arg-BODIPY molecules or transient interactions with the membrane. We also investigated possible fluorescence emission changes of fluorescently labeled AMs (10 μM) when incubated with LUVs (0.5 mg/mL) for 15 min (Figure 2C,D, Table 1). All AMs displayed a highly significant increase in fluorescence emission when incubated with LUVs, confirming their ability to bind to LUVs (*p* < 0.001). Notably, SQ labeled with both dyes showed a greater increase compared to the other two AMs, as noticed also for the *r* value of SQ-A594 (*p* < 0.001). This can be attributed to the shorter polyamine group of SQ relative to TRO and ENT-03, which reduces the distance of the dye probe from the membrane. Fluorescence of 10 μM L-Arg-BODIPY and L-Arg-A594 did not change significantly upon LUV addition (Figure 2B,C, Table 1).

**Table 1:** Fluorescence Anisotropy and Intensity of AMs Incubated with LUVs^a^

AM	*r* (− LUVs)	*r* (+ LUVs)	fluorescence (− LUVs) (a.u.)	fluorescence (+ LUVs) (a.u.)	*K*_D_ (mg/mL)	*K*_D_ (μM)
L-Arg-BODIPY	0.0155 ± 0.0004	0.0595 ± 0.0016	3994 ± 8	3964 ± 87		
TRO-BODIPY	0.0271 ± 0.0008	0.2173 ± 0.0033	4010 ± 8	8828 ± 154	0.0302 ± 0.0032	38.7 ± 4.1
SQ-BODIPY	0.0289 ± 0.0009	0.2221 ± 0.0038	4136 ± 51	19,358 ± 635	0.0169 ± 0.0023	21.6 ± 2.9
ENT-03-BODIPY	0.0258 ± 0.0010	0.2296 ± 0.0017	3994 ± 15	6435 ± 201	0.0321 ± 0.0064	41.1 ± 8.2
L-Arg-A594	0.0294 ± 0.0007	0.0302 ± 0.0007	4085 ± 7	4141 ± 63		
TRO-A594	0.0493 ± 0.0009	0.1630 ± 0.0020	4483 ± 29	8471 ± 779	0.1516 ± 0.0545	195 ± 70
SQ-A594	0.0440 ± 0.0025	0.2349 ± 0.0014	4678 ± 23	16,612 ± 1431	0.0620 ± 0.0093	79 ± 12
ENT-03-A594	0.0471 ± 0.0014	0.1301 ± 0.0057	4541 ± 38	7304 ± 362	0.1164 ± 0.0603	148 ± 81

a Anisotropy (*r*) at 570 and 617 nm and intensity area between 550–650 and 600–700 nm for BODIPY and A594-labeled L-Arg and AMs, respectively, in the absence and in the presence of 0.5 mg/mL LUVs. *K*_D_ values obtained from the binding experiments expressed in mg/mL and μM of total lipids. Experimental errors are standard error of the mean (SEM) of *n* = 5 technical replicates.

### SQ Exhibits the Highest Affinity among the Three AMs for LUVs

**Figure 3:**
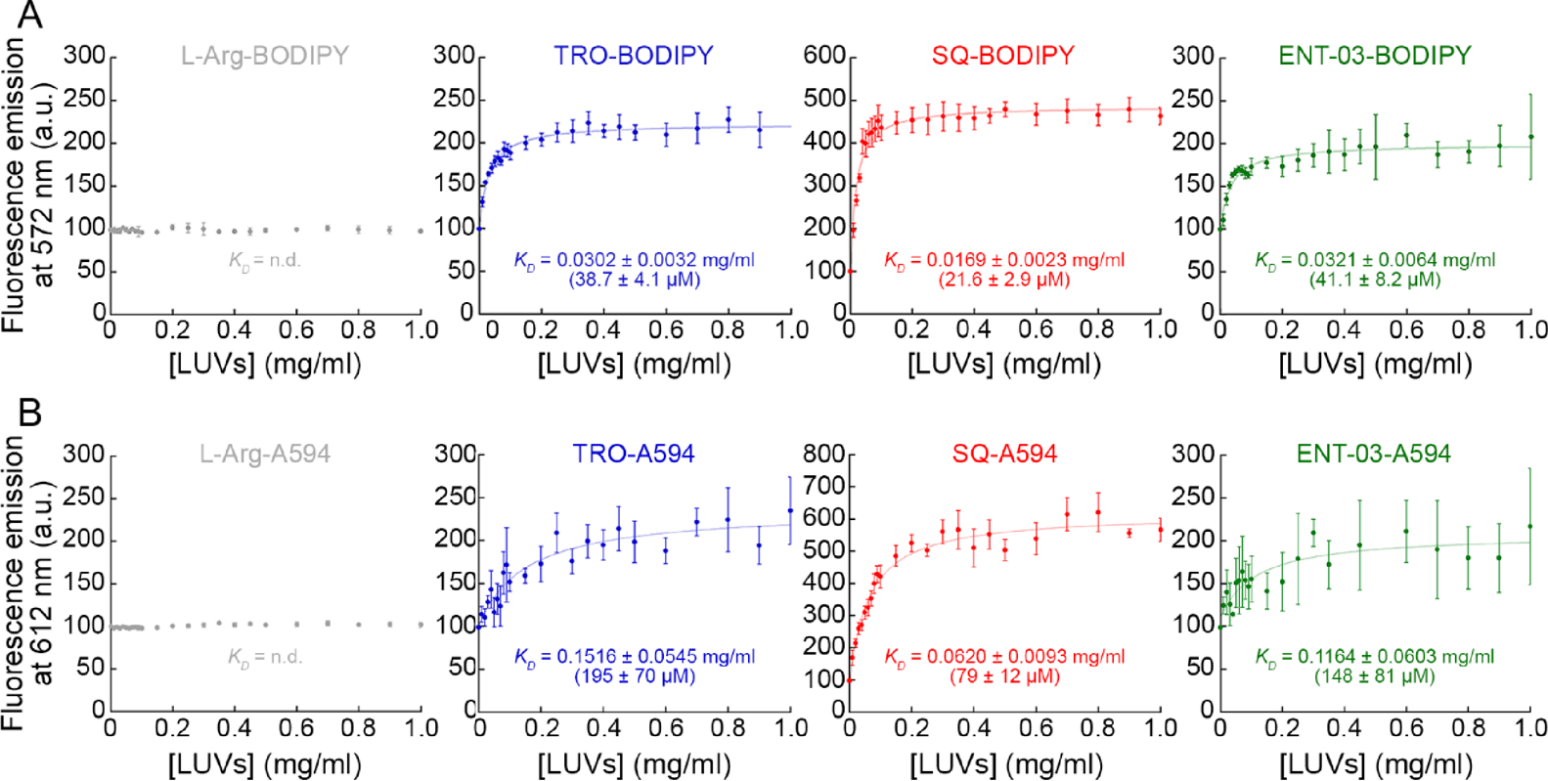
Binding of the three AMs to LUVs. Binding plots reporting the fluorescence emission at 572 nm (A) and 612 nm (B), of 10 μM BODIPY (A) and A594 (B) labeled TRO (blue), SQ (red), ENT-03 (green), and L-Arg (gray) versus LUV concentration. The lines through the data points represent the best fits to eq 6. Each graph reports the obtained *K*_D_ value in units of mg/mL and μM of total lipids. Experimental errors are SEM (*n* = 5).

Since all three labeled AMs increase their fluorescence upon LUV binding, we exploited this spectroscopic property to obtain quantitative measurements of the affinity of the three labeled AMs for LUVs. They were therefore incubated for 15 min (10 μM) with increasing concentrations of unlabeled LUVs and we observed in all cases a significant increase in fluorescence emission (Figure 3). By fitting the data points to a standard binding curve (eq 6), it was possible to obtain *K*_D_ values of all labeled AMs (Figure 3, Table 1). With both fluorescent dyes, SQ confirmed the highest increase of fluorescence upon LUV binding and also showed a *K*_D_ value approximately 2-fold lower relative to the other two AMs, (*p* < 0.05), indicating a higher affinity for LUVs compared to the other two AMs. This observation can be attributed to the shorter hydrophilic spermidine moiety of SQ, as opposed to the longer hydrophilic spermine moiety of TRO and ENT-03, which increases the overall hydrophobicity of SQ and explains its higher affinity for LUVs. By contrast, TRO and ENT-03 displayed similar *K*_D_ values in both analyses with BODIPY and A594 (*p* = 0.8198 and *p* = 0.7380, respectively, nonsignificant).

The *K*_D_ value determined with BODIPY for a given AM was about 4-fold lower than that determined for the same AM with A594, indicating that the chemistry of the probe affects the AM-LUV affinity to some extent. However, the rankings and relative differences of the *K*_D_ values determined for the three AMs are similar when determined with either probe, indicating in both cases that TRO and ENT-03 have similar affinities for the LUV membrane, within experimental error, and that SQ has a ca. 2-fold higher affinity. Since the two probes add a hydrophilic and hydrophobic component to the AMs, it is likely that the binding constant of a given unlabeled AM is intermediate between these two values. Negative controls with labeled L-Arg and increasing concentrations of LUVs showed the lack of variation in fluorescence, confirming that the binding abilities of labeled AMs to LUVs were mediated by the AM rather than the fluorescent probe bound to them (Figure 3).

To explore the kinetics of the AM-LUV binding, TRO-A594 as a representative AM and LUVs were rapidly mixed using a stopped-flow apparatus to final concentrations of 10 μM and 0.5 mg/mL, respectively, and the TRO-A594 fluorescence change during the binding process was monitored in real time (Figure S1A). Two kinetic phases were observed, occurring on the time scales of ca. 500 ms and 10 s, respectively, therefore indicating that the binding was very rapid and that, after a time of 15 min explored here, binding has attained equilibrium. This holds true even at the lowest LUV concentration (0.12 mg/mL) tested (Figure S1B,C). These two phases may either represent the signature of a two-step binding mechanism or reflect two ligand subpopulations that bind LUVs with different kinetics. Assignment of the two phases to well defined molecular events is beyond the scope of the present analysis.

### SQ Exhibits the Lowest Occupancy within LUVs among the Three AMs

**Figure 4:**
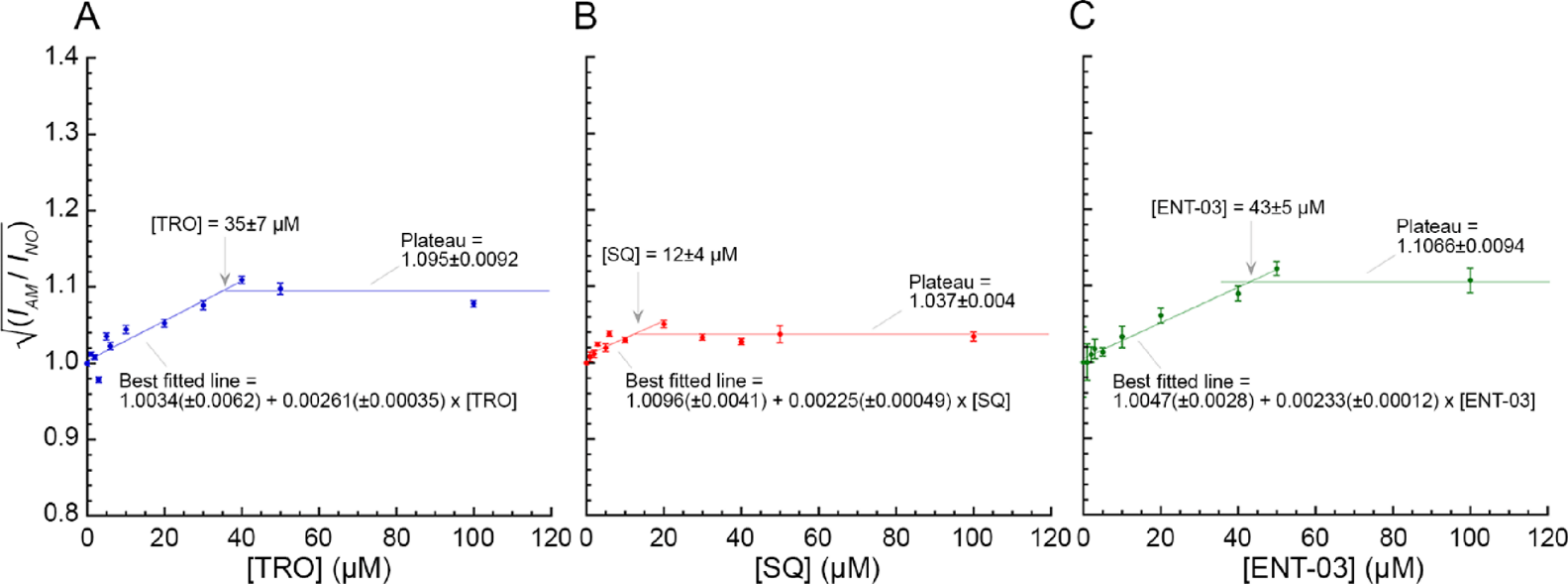
Light scattering intensity of LUVs in the presence of increasing concentrations of the three AMs. Plots reporting the square root of the light scattering intensity of LUVs with (*I*_AM_) and without (*I*_NO_) AM, respectively, versus AM concentration, representing the increase in LUV mass due to TRO (A), SQ (B), and ENT-03 (C) incorporation. The indicated AM concentrations correspond to the values reported on the *x* axis at saturation points. Experimental errors are SEM (*n* = 3).

The steric hindrance and chemical properties of the dyes bound to AMs could in principle have an impact on the interaction of AMs with LUVs. Therefore, we sought an additional experimental approach to probe the incorporation of TRO, SQ, and ENT-03 in their free unlabeled form with LUVs. To this aim, a light scattering analysis using only unlabeled species was carried out (0–100 μM AMs, 0.5 mg/mL LUVs, 15 min). Since light scattering intensity is proportional to the second power of the mass of the light scattering particles, by incubating LUVs with increasing concentrations of the three AMs, it was possible to obtain a measure of the increase of LUV mass due to AM incorporation in these three cases (Figure 4).

All AMs induced an increase in light scattering intensity, and thus LUV mass, until a saturating concentration, after which they exhibited a plateau phase, where no more AMs were incorporated. SQ reached the plateau at a significantly lower concentration compared to the other AMs (Figure 4B, *p* < 0.05), whereas TRO and ENT-03 displayed similar saturating concentrations (Figure 4A,C, *p* > 0.05). The saturating concentrations were found to be 35 ± 7, 12 ± 4, and 43 ± 5 μM for TRO, SQ, and ENT-03, respectively. These values can also be obtained from the LUV mass increase at saturation, which yields the mass of incorporated AMs, which were found to be 35 ± 4, 15 ± 2, and 43 ± 4 μM for TRO, SQ, and ENT-03, respectively, in very good agreement with those estimated above. All AMs did not show a significant increment of LUV diameter (Figure S2), ruling out that the observed increase of light scattering intensity upon AM addition was due to an increase in LUV size. This analysis indicates that AMs bind to LUVs even without fluorescent labeling and allows the maximum AM occupancy to be estimated for all three AMs.

To obtain an independent estimate of the AM occupancy at LUVs at saturation, we employed a microfluidic technique using LUVs and TRO as a representative AM (Figure S3). We obtained a value of ca. 28–35 μM TRO at saturation, in agreement with the value of 35 ± 4 μM TRO estimated with light scattering (Figure S3).

### All Three AMs Partially Neutralize the Negative Charge of LUVs, with an Efficacy TRO ≅ ENT-03 > SQ

Since 5 μM AM and 1.0 mg/mL LUVs are concentrations at which binding is complete (all AM is bound to LUVs, and LUVs are not yet saturated), we carried out the following analyses at these concentrations, in all cases after covesiculating AMs with the lipids of LUVs to rule out incomplete binding. In particular, we evaluated the effect of the three AMs on three physicochemical properties of the LUV lipid bilayer previously found to be altered by TRO and thought to represent important factors for the vulnerability of the lipid plasma membrane to misfolded protein oligomers:^11^ the membrane negative charge, monitored with zeta potential (ζ) measurements, the resistance of the bilayer to a breakthrough force (BTF) perpendicular to the bilayer plane, monitored with supported lipid bilayers (SLBs) and atomic force microscopy (AFM), and the distribution of lipids in the membrane, monitored with lipid–lipid fluorescence resonance energy transfer (FRET).

**Figure 5:**
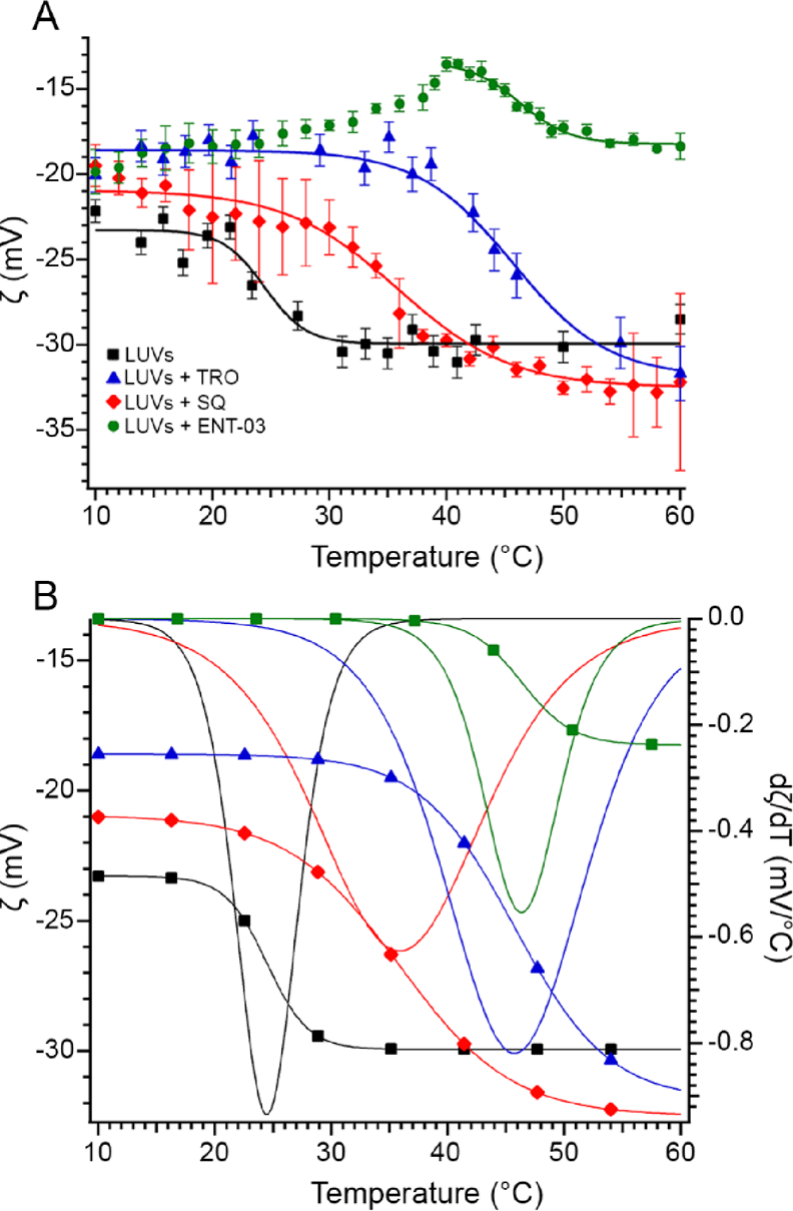
The three AMs increase the transition temperatures of LUVs. (A) Zeta potential (ζ) and (B) fitted curves (left axis) and first derivative curves (right axis) of ζ values as a function of temperature for naked LUVs (black square), and AM-containing LUVs: TRO (blue), SQ (red), and ENT-03 (green). Experimental errors are standard deviations (*n* = 5). In each case, the *T*_m_ corresponds to the minimum of the first derivative curve.

The ζ was measured using naked LUVs and LUVs covesiculated with TRO, SQ, or ENT-03 (Figure 5A, Table 2). For naked LUVs, a negative value of ζ of −23.6 ± 0.7 mV was found at 20 °C,^11^ whereas values of −18.7 ± 0.3, −21.0 ± 0.5, and −18.7 ± 0.3 mV (mean ± SEM) were obtained for LUVs covesiculated with TRO, SQ, and ENT-03, respectively, at the same temperature (Figure 5A, Table 2). These variations indicate, in all cases, a partial neutralization of the total negative surface charge of LUVs, or molecular packing of the charged lipid heads, upon AM addition (*p* < 0.001, *p* < 0.05, and *p* < 0.001, respectively). SQ induced a smaller decrease compared to TRO and ENT-03 (*p* < 0.01), which showed by contrast comparable decreases (*p* > 0.05). This is in agreement with the chemical properties of the three AMs, with SQ carrying a spermidine group, which is shorter and less positively charged than the spermine group of TRO and ENT-03.

**Table 2:** Experimental Values of the Physicochemical Perturbations of the Membrane Induced by AMs^a^

Parameter	ζ (mV)	Δζ (%)	BTF (nN)	ΔBTF (%)	*r*_GM1-CHOL_ (Å)	Δ*r*_GM1-CHOL_ (%)	*r*_CHOL-CHOL_ (Å)	Δ*r*_CHOL-CHOL_ (%)
– AMs	–23.6 ± 0.7	0%	2.73 ± 0.09	0%	65 ± 1	0%	72 ± 1	0%
+ TRO	–18.7 ± 0.3	100%	4.2 ± 0.2	100%	79 ± 1	100%	58 ± 1	100%
+ SQ	–21.0 ± 0.5	53%	3.5 ± 0.2	52%	72 ± 1	50%	64 ± 1	57%
+ ENT-03	–18.7 ± 0.3	100%	3.0 ± 0.1	18%	73 ± 1	57%	63 ± 2	64%

a Zeta potential (ζ), breakthrough force (BTF), GM1/CHOL, and CHOL/CHOL mean shortest distance (*r*) experimental values, and their corresponding normalized percent values were obtained in the absence and presence of TRO, SQ, and ENT-03. BTF errors were evaluated as half of the range of variability of mean values from different series of measurements for each condition. Experimental *r* errors are SEM (*n* = 3 technical replicates). ζ potential errors are standard deviations (*n* = 5).

ζ measurements versus temperature were then used to determine the phospholipid gel to liquid-crystalline phase transition temperature (*T*_m_) of LUVs in the absence and presence of each AM (Figure 5A,B). The transition is described by a sharp change in the ζ potential (Figure 5A) that is more evident in corresponding first derivative plots (Figure 5B). The *T*_m_ of this LUV system is dominated by SM (*T*_m_ of 35–40 °C), since DOPC (*T*_m_ of −17 °C), which is the most abundant lipid, is already in a fluid-like phase in the examined temperature range.^19,20^ For naked LUVs, the *T*_m_ was observed at 24.5 ± 2 °C. All AMs induced an increase of the transition temperature of LUVs, up to values of 46 ± 2 °C (*p* < 0.01), 35 ± 2 °C (*p* < 0.05), and 46 ± 2 °C (*p* < 0.01) with LUVs covesiculated with TRO, SQ, and ENT-03, respectively, indicating trend variations similarly to those measured with ζ at 20 °C. The transition width is about 6 °C for the naked LUVs, but becomes larger with AM, up to 16 °C in the case of TRO-containing LUVs, confirming that AMs modify the packing of the bilayer and system disorder.^21^

### All Three AMs Increase the Breakthrough Force of Supported Lipid Bilayers, with an Efficacy of TRO > SQ > ENT-03

**Figure 6:**
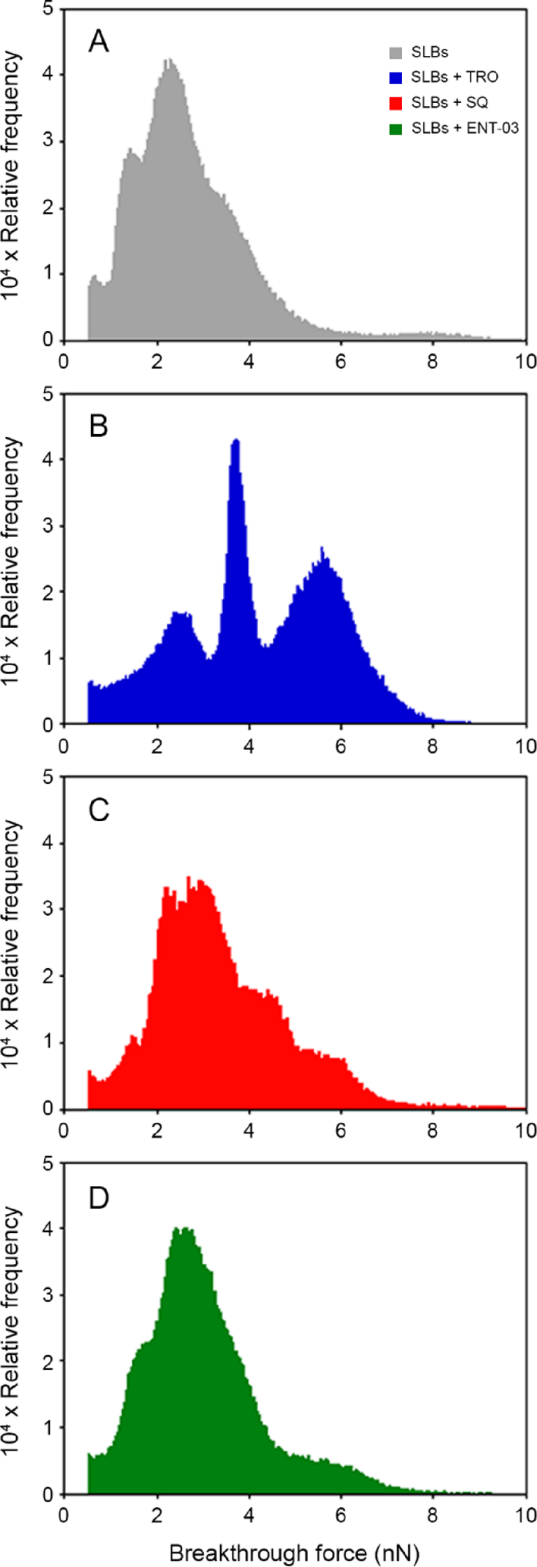
The three AMs increase the mechanical resistance of lipid bilayers to indentation or BTF. Breakthrough force (BTF) distributions measured on SLBs formed from LUVs prepared in the absence (A, gray) and in the presence of 5 μM TRO (B, blue), SQ (C, red), and ENT-03 (D, green). Distributions were obtained from at least six independent force maps. The statistically significant difference between AMs was calculated using a Kruskal-Wallis test, which resulted in *p* < 0.001, and a Dunn test, which highlighted a difference between each group with *p* < 0.05.

The resistance of the bilayer to a force applied perpendicular to its plane was analyzed by AFM, which allowed us to determine the breakthrough force (BTF) required to penetrate the bilayer with the AFM tip. Measurements were performed on SLBs in the absence and presence of each AM (Figure 6). As previously observed, SLBs with this lipid composition display the coexistence of two different phases: gel-phase domains (Lβ or So), with possible contributions of liquid-ordered domains (Lo), enriched with SM, CHOL, and GM1, that float in a liquid-disordered phase (Lα or Ld) enriched with DOPC.^11,22^ The largest fraction of breakthroughs was observed on Lα regions, while most of the Lβ regions displayed the absence of breakthrough events.^11^ The presence of AMs determined an increase in BTF values relative to AM-devoid SLBs (BTF of 2.73 ± 0.09 nN; Table 2). TRO caused the largest increase, followed by SQ and then ENT-03, with BTF values of 4.2 ± 0.2, 3.5 ± 0.2, and 3.0 ± 0.1 nN, respectively (Figure 6, Table 2).

### All Three AMs Redistribute Cholesterol and GM1 Lipids in LUVs, with Efficacy TRO > SQ ≅ ENT-03

**Figure 7:**
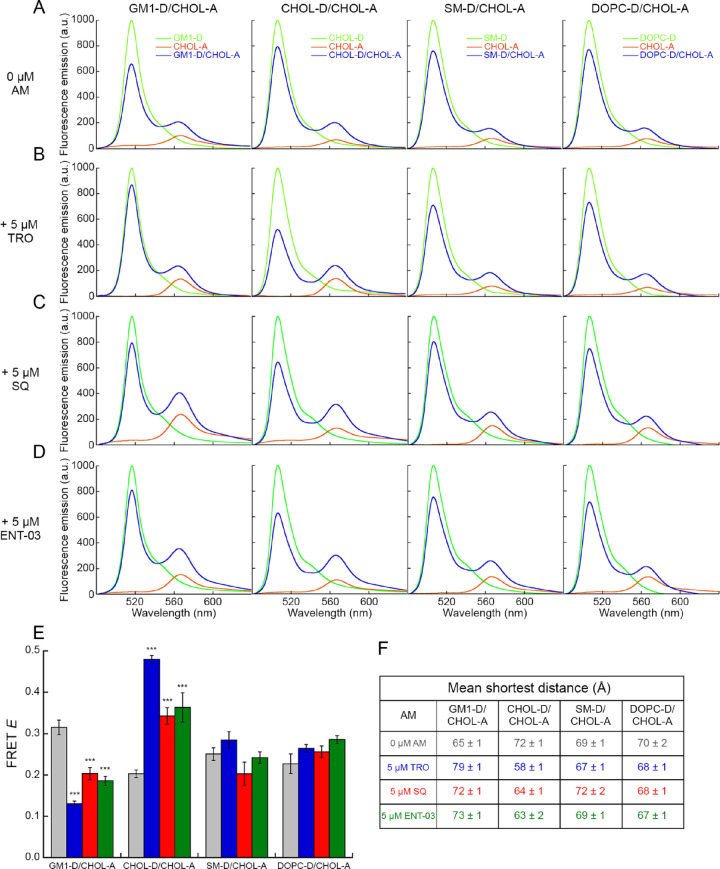
The three AMs redistribute CHOL and GM1 molecules in LUVs. (A–D) Fluorescence spectra of LUVs containing the indicated D-labeled lipid (green), A-labeled CHOL (orange), and both (blue) in the absence (A) and in the presence of TRO (B), SQ (C), and ENT-03 (D). (E) FRET efficiency (*E*) values obtained for the various pairs using eq 12 in the absence (gray) and presence of TRO (blue), SQ (red), and ENT-03 (green). Experimental errors are SEM (*n* = 5). The symbols *** refer to *p* values of <0.001 relative to *r* values obtained in the absence of AMs. (F) Mean shortest distances (*r*) between the indicated lipid-D and CHOL-A in absence and presence of AMs obtained from FRET *E* values reported in panel E using eq 13.

Using lipid-lipid FRET, TRO was previously found to reorganize the spatial distribution of CHOL and GM1 molecules in LUVs, therein clustering CHOL molecules, separating CHOL from GM1 molecules, clustering GM1 molecules, and maintaining mutual distances of SM and DOPC from CHOL.^11^ To investigate whether SQ and ENT-03 could have a similar impact, we carried out a series of lipid–lipid FRET experiments in the absence and presence of each AM. In these experiments, 0.0625% of a given lipid (relative to the total lipid content in LUVs) was labeled with a donor (D) fluorescent probe and the same fraction of CHOL was labeled with an acceptor (A) fluorescent probe, namely, BODIPY FL and BODIPY 542/563, respectively. Four different combinations of FRET pairs (GM1-D/CHOL-A, CHOL-D/CHOL-A, SM-D/CHOL-A, and DOPC-D/CHOL-A) were then analyzed in the presence of SQ and ENT-03 and then compared to data obtained with LUVs without AMs and with LUVs with TRO (Figure 7A–D). Both SQ and ENT-03 induced a similar and significant increment of FRET efficiency (*E*) in the CHOL-D/CHOL-A pair (*p* < 0.001) and a reduction in the GM1-D/CHOL-A pair (*p* < 0.01), indicating a reduction of the mean shortest distance between CHOL molecules or GM1 molecules and an increase of the distance between CHOL and GM1 molecules (Figure 7E,F, Table 2). This behavior was similar to that induced by TRO (*p* < 0.001 relative to absence of AM), but was found to occur to a significantly lesser extent (Figure 7E,F, Table 2). Neither SQ nor ENT-03 showed variations in the FRET *E* for the SM-D/CHOL-A and DOPC-D/CHOL-A pairs (*p* > 0.05), in agreement with the effect of TRO (Figure 7E,F, Table 2).

### All Three AMs Displace α-Synuclein from DMPS LUVs, with Efficacy TRO ≅ ENT-03 > SQ

**Figure 8:**
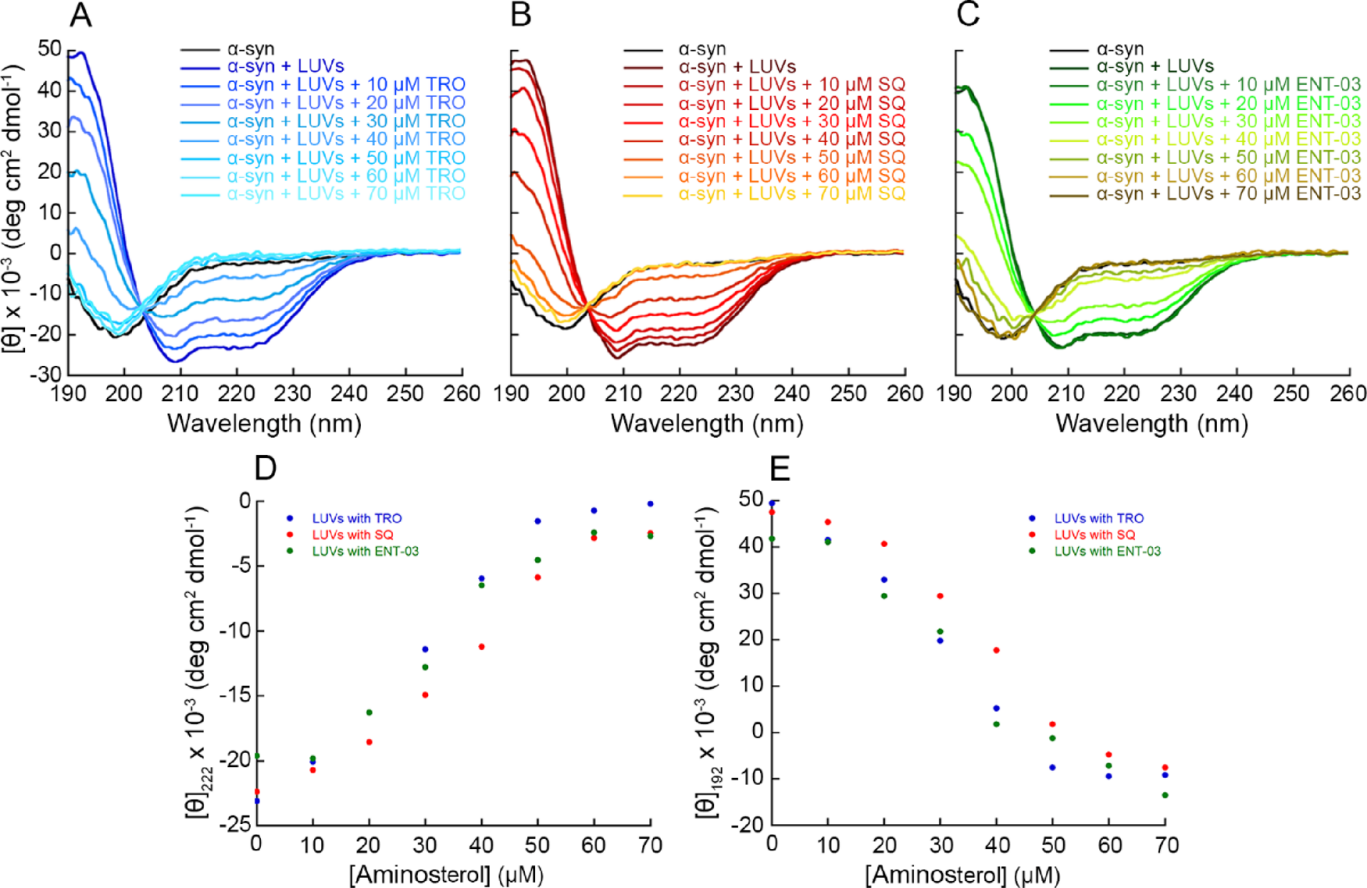
Far UV CD analysis of α-synuclein displacement exerted by the three AMs. (A–C) Far-UV CD spectra of αS in the absence and presence of DMPS LUVs incubated with increasing concentrations of TRO (A), SQ (B), and ENT-03 (C). Spectra were blank subtracted and normalized using eq 14. (D, E) Mean residue ellipticity at 222 nm (D) and 192 nm (E) of αS incubated with DMPS LUVs and increasing concentrations of TRO (blue), SQ (red), and ENT-03 (green).

Previous works showed that TRO and SQ were able to displace α-synuclein (αS) from small unilamellar vesicles (SUVs) composed of DMPS,^4,5^ and that this displacement could be monitored as a change from a LUV-bound αS conformation enriched with an α-helical structure and free, unbound, substantially disordered αS conformational state.^4,5^ We repeated the experiments with TRO and SQ and also extended the analysis to ENT-03, using DMPS LUVs rather than our ordinary LUVs to replicate the previously established protocol^4,5^ and because the strength of the binding of αS to lipids is strongly influenced by the chemical properties of the lipids.^23−25^ To this aim, 5 μM αS was incubated with 0.2 mg/mL DMPS LUVs for 30 min and then with increasing AM concentrations for 15 additional min. All AMs were found to displace αS from LUVs in a dose-dependent manner, as shown by the progressive apparent two-state change from a typical α-helical CD spectrum, with negative peaks at 222 and 208 nm and a positive peak at 192 nm, to a typical random-coil spectrum, with a single negative peak at 198 nm (Figure 8A–C).

This conformational change was compared for the three AMs as a progressive increase of mean residue ellipticity at 222 nm, showing that SQ is slightly less effective, as it requires higher concentrations to displace the protein from the membrane relative to TRO and ENT-03, which had similar displacement activities (Figure 8D). This ranking suggests that the AM-induced displacement of αS from DMPS LUVs is mainly driven by electrostatic effects, given that TRO and ENT-03 are more positively charged than SQ at physiological pH.

### All Three AMs Protect the Plasma Membrane of Cultured Cells from Aβ Oligomers, with Efficacy TRO > ENT-03 > SQ

**Figure 9:**
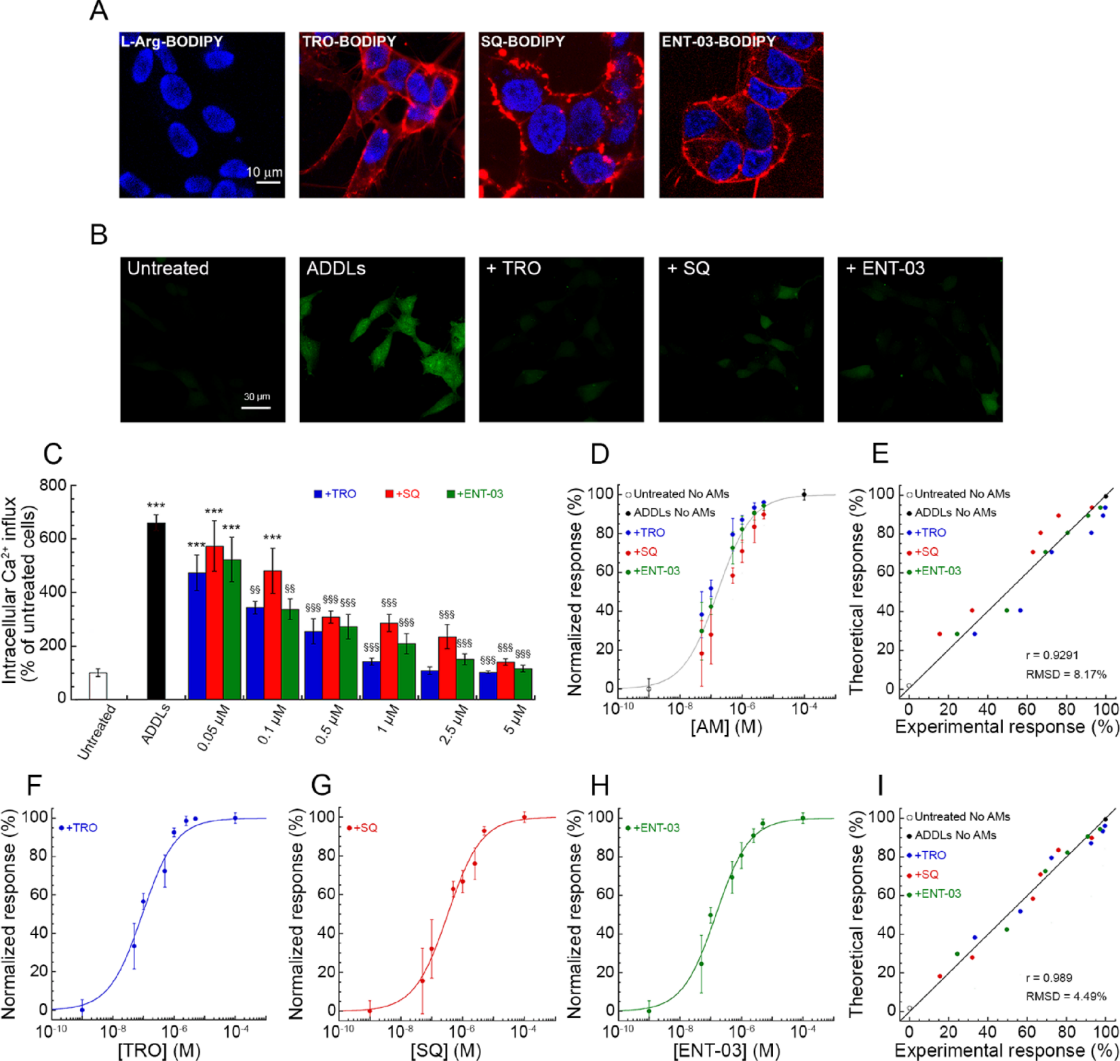
The three AMs bind to the plasma membrane of SH-SY5Y cells and prevent the increase of intracellular Ca^2+^ levels induced by ADDLs (global fitting analysis). (A) Representative confocal microscopy images (median planes parallel to the coverslip) of SH-SY5Y cells incubated for 30 min at room temperature with 5 μM of L-Arg-BODIPY, TRO-BODIPY, SQ-BODIPY, or ENT-03-BODIPY (probe:molecule of 1:10). Blue and red fluorescences indicate Hoechst-labeled nuclei and AM-BODIPY, respectively. (B) Representative confocal scanning microscopy images of free Ca^2+^ levels in untreated SH-SY5Y cells or in cells treated for 15 min with 1 μM ADDLs in the absence or presence of 1 μM AMs. (C) Intracellular free Ca^2+^-derived fluorescence in untreated SH-SY5Y cells or in cells treated for 15 min with ADDLs in the absence or presence of the indicated concentrations of AMs. Experimental errors are SEM (*n* = 4). *** symbols refer to *p* values <0.001 relative to untreated cells. §§ and §§§ symbols refer to *p* values <0.01 and < 0.001, respectively, relative to ADDLs without AMs. (D) Normalized dose–response curve obtained from Ca^2+^-derived fluorescence data of all AMs in panel C and fitted to eq 2. (E) Plot reporting theoretical versus experimental response values obtained from eq 2. (F–H) Normalized dose–response curves for TRO (blue), SQ (red), and ENT-03 (green) obtained from Ca^2+^-derived fluorescence data in panel C. In each plot, the lines through the data do not represent independent fitting procedures using eq 2, but result from global fitting using only eq 4, in all cases with corresponding values of ζ potential, BTF, and FRET data. Experimental errors are SEM (*n* = 4). (I) Plot reporting theoretical versus experimental response values obtained from eq 4.

We then passed from LUVs to cultured cells (human neuroblastoma SH-SY5Y cells) to investigate whether all three AMs bind to the plasma membrane of cells and protect them from misfolded protein oligomers. All three AMs were labeled with BODIPY TMR, which is a neutral and hydrophobic probe that does not alter the positive net charge of the AM. SH-SY5Y cells were treated with 5 μM TRO-BODIPY, SQ-BODIPY, or ENT-03-BODIPY for 30 min at room temperature and analyzed with confocal scanning microscopy (Figure 9A). All three AMs prominently bind to the plasma membrane, in accordance to the results obtained with LUVs. Moreover, cells treated with L-Arg-BODIPY, used here as a negative control, show the total absence of BODIPY-derived fluorescence (Figure 9A), confirming that the binding observed using AM-BODIPY is fully attributable to the AM, rather than the hydrophobic probe.

The protective effect of the three AMs against the ability of misfolded protein oligomers to cause cell dysfunction was evaluated using amyloid-β-derived diffusible ligands (ADDLs) composed of Aβ_42_ as sample oligomers and evaluating the influx of calcium ions (Ca^2+^) from the extracellular space to the cytosol of cultured SH-SY5Y cells, which is thought to be the earliest insult following the oligomer-membrane interaction.^26−30^ SH-SY5Y cells were treated for 15 min with ADDLs (1 μM, monomer equivalents) in the absence or presence of different concentrations of AMs and then their Ca^2+^ levels were evaluated with a specific fluorescent probe that enters inside the cells and produces green fluorescence (*F*) only when bound to Ca^2+^ (Figure 9B,C). ADDLs caused a 660 ± 30% increase of Ca^2+^ relative to untreated cells, indicating a markedly heightened state of toxicity. Coincubation of the ADDLs with AMs caused a decrease of Ca^2+^ levels with a clear dose-dependence and AM-type dependence. In particular, TRO was found to be more effective than ENT-03 at corresponding concentrations, and the difference was statistically significant when all doses were analyzed together (*p* < 0.01). TRO and ENT-03 were both more effective than SQ (*p* < 0.001 in both cases). At the highest AM concentration tested (5 μM), all three AMs were able to completely suppress the ADDL-induced Ca^2+^ influx down to the levels of untreated cells (Figure 9C).

### Global Fitting Analysis Determines Quantitatively the Chemical Factors of AMs and the Physico-Chemical Determinants of Membrane Involved in the AM-Induced Membrane Protection in this Experimental Setting

All data of Ca^2+^-derived fluorescence (*F*) shown in Figure 9C were converted into normalized response (*R*) values ranging from 0% (no effect) to 100% (full effect) using:
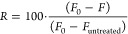
1where *F*_0_ and *F*_untreated_ are the *F* values with and without ADDLs, respectively, both without AMs (corresponding to 659 ± 30 and 100 ± 15%, respectively). The obtained *R* values were plotted versus AM concentration in one single semi-log plot to obtain the typical dose–response curve used in pharmacology (Figure 9D). The resulting plot was then fitted to the Hill equation, typically used to analyze dose–response curves and found to satisfactorily fit most dose–response curves:^31^
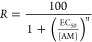
2where [AM] is the AM molar concentration, EC_50_ is the molar AM concentration at which *R* was 50%, and *n* is the Hill coefficient. EC_50_ and *n* were parameters free to float in the fitting procedure, and values of 1.62 ± 0.25 × 10^–7^ M and 0.78 ± 0.09 were obtained, respectively (Figure 9D, *r* = 0.9291, RMSD = 8.17%). A fairly good agreement was found between theoretical *R* values redetermined with eq 2 for all three AMs and corresponding experimental *R* values (Figure 9E, *r* = 0.9291, RMSD = 8.17%). Nevertheless, the agreement was not entirely satisfactory due to differences among the three AMs.

To improve the agreement and identify the AM-induced membrane alterations responsible for the observed changes of *R* values at corresponding AM concentrations, we recognize two different contributions to the EC_50_ of the AMs: the change in charge and the change in packing, which add to an offset EC_50_ value in the absence of these two changes (EC′_50_):
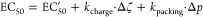
3

The two contributions were considered additive, in the absence of knowledge of a well-defined relationship, as generally done in empirical equations.^32^ This leads to a phenomenological Hill equation, where all *R* values were analyzed in a multivariable and multiparameter global fitting procedure:
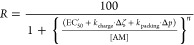
4where Δζ is the experimentally determined and normalized change of ζ observed upon AM addition (corresponding to the percent values reported in Table 2); Δ*p* is the experimentally determined and normalized change of lipid distribution and packing observed upon AM addition (corresponding to the averaged three remaining percent values reported in Table 2); *k*_charge_ and *k*_packing_ are the proportionality constants for Δζ and Δ*p*, respectively; EC′_50_ is a parameter corresponding to EC_50_ when Δζ and Δ*p* are both equal to 0 and corresponds to the EC_50_ for a hypothetical nonnatural AM that does not affect ζ and *p*; and *n* has the same meaning described for eq 2.

This equation represents a Hill equation integrated with the *k*_charge_·Δζ and *k*_packing_·Δ*p* factors. Its three independent variables ([AM], Δζ, and Δ*p*) are used to express one dependent variable (*R*), upon the global fitting of the four constants (EC′_50_, *k*_charge_, *k*_packing_, and *n*) using all the available data. Fitting all *R* values to eq 4 yielded values of 5.94 ± 0.59 × 10^–7^ M, −3.99 ± 0.82 × 10^–7^, −1.03 ± 0.22 × 10^–7^, and 0.795 ± 0.015 for EC′_50_, *k*_charge_, *k*_packing_, and *n*, respectively. The equation can describe well the behavior of the three AMs plotted separately in three independent graphs (Figure 9F–H), where the three solid lines through the data do not represent the results of three independent fitting procedures, but are rather the result of one equation determined from the global fitting. A very good and improved agreement was found between theoretical *R* values redetermined with eq 4 for all three AMs and the corresponding experimental *R* values (Figure 9I, *r* = 0.9890, RMSD = 4.49%).

The model and resulting eq 4 were validated using the leave-one-out cross-validation (LOOCV) method, in which each experimental *R* value was left out from the analysis, one by one, to redetermine, through the global fitting, the four constants of eq 4 and the resulting theoretical *R* value corresponding to the left-out experimental *R* value. A good agreement was found between redetermined theoretical versus experimental *R* values (Figure S4, *r* = 0.982, RMSD = 5.70%), indicating the ability of eq 4 to predict new *R* values that are not present in the analysis.

What can we learn from this analysis? The *k*_charge_ and *k*_packing_ values are both negative, indicating that both the partial charge neutralization and compaction of the membrane contribute to the increase of AM potency (corresponding to a decreased EC_50_ value). Their relative contributions amount to 79 ± 7 and 21 ± 7%, respectively, indicating that the charge effect is predominant. A hypothetical nonnatural AM that does not affect ζ and *p* (for example, having a monoamine group and shorter tail on the other side) would have a potency 6–7-fold lower than that of TRO. TRO appears the most effective AM because it produces the highest effects in terms of both membrane neutralization and compaction. ENT-03 is marginally, albeit significantly, less effective because it has a much lower effect on membrane compaction. This feature accounts for only 21 ± 7% of the effect, therein explaining why its potency is only slightly lower. By contrast, SQ appears markedly less effective because it leads to a much lower change in charge than TRO and ENT-03, despite a packing effect similar to that of ENT-03.

We also included the different binding affinities (*K*_D_) of the AMs for LUVs as a parameter in the global fitting, but this has not resulted in any improvement, particularly because the least protective SQ has also the highest binding affinity for the lipid bilayer, suggesting that binding affinity is a less relevant factor. This can be rationalized by the fact that the lipid concentration is very high in the two-dimensional carpets of cells, making the AMs work in a saturation regime similar to that of high LUV concentration in the conditions explored in Figure 3. On different grounds, AM occupancy on the membrane is also not a factor because all AM concentrations tested here on cells (0–5 μM) are well below the AM concentrations determined experimentally at saturation on LUVs (12–50 μM), and the latter are certainly even higher in cell cultures.

### Potency and Membrane Perturbations Can Be Attributed to Specific AM Chemical Groups

To attribute the potency as well as the *k*_charge_ and *k*_packing_ parameters and their numerical values to discrete chemical groups within the AMs, we repeated the analysis using modifiers of the Hill equation that account for chemical differences between the three AMs (*a*_polyamine_ and *a*_SO3/COO_) rather than experimental observables (Δζ and Δ*p*):
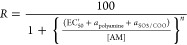
5where [AM] and *R* are the independent and dependent variables, respectively; EC′_50_, *a*_polyamine_, and *a*_SO3/COO_ are constants free to float in the global fitting analysis using all the available data. *a*_polyamine_ and *a*_SO3/COO_ are parameters accounting for the change of the EC′_50_ parameter when a spermine, rather than a spermidine, and a −SO_3_^–^, rather than a −COO^–^ group, are present in the AM, respectively. The two parameters were constrained to 0 when the spermine and −SO_3_^–^ groups were absent and replaced by spermidine and carboxylate, respectively. Fitting all *R* values to eq 5 yielded values of 3.83 ± 0.39 × 10^–7^, −2.36 ± 0.47 × 10^–7^, and −0.55 ± 0.12 × 10^–7^ M for EC′_50_, *a*_polyamine_, and *a*_SO3/COO_, respectively (*r* = 0.9820, RMSD = 4.50%).

The *a*_polyamine_ and *a*_SO3/COO_ values were again found to be negative, indicating that the longer spermine and SO_3_^–^-containing tail cause a potency increase (or EC_50_ decrease) relative to a hypothetical nonnatural AM having a spermidine and carboxylate group. They account for 81 ± 7 and 19 ± 7% of the effect, respectively, in good agreement with the values obtained with the experimental analysis and confirming that the charge effect of the polyamine is predominant over the chemistry of the tail on the other side of the molecule that causes a redistribution of lipids and increased packing. The net positive charge of AMs allows a larger decrease of the negative charge of the cell membrane provided by GM1, which is a physicochemical change reported to be crucial in protecting biological membranes from Aβ oligomer binding and resulting cell toxicity also using other compounds, such as europium positive ions.^33^ The EC′_50_ value obtained for the hypothetical nonnatural AM with spermidine and carboxylate (3.83 ± 0.39 × 10^–7^ M), which is higher than that of any AM analyzed here having at least the spermine (triamine) or SO_3_^–^ group, is lower than the hypothetical nonnatural AM of the previous analysis having a monoamine group and shorter tail on the other side (5.94 ± 0.59 × 10^–7^ M). Since all natural and nonnatural AMs studied here have a steroid scaffold, their EC_50_ values remain in the 10^–8^–10^–7^ M range.

## Conclusions

The three natural AMs studied here have a different chemistry and combination of specific, relevant functional groups. Consequently, their binding to the lipid bilayer of a liposome membrane results in different perturbations of the physico-chemical properties of the liposome bilayer, such as their surface charge (ζ), resistance to a mechanical breakthrough force (BTF) perpendicular to its plane and distribution of CHOL, and GM1 lipids (*r*). Importantly, their binding to the membranes of cultured SH-SY5Y cells results into different degrees of protection against the action of misfolded protein oligomers of the Aβ_42_ peptide to Ca^2+^ influx. The protection depends on AM concentration, level of charge neutralization of the membrane and change of packing resulting from lipid redistribution.

**Figure 10:**
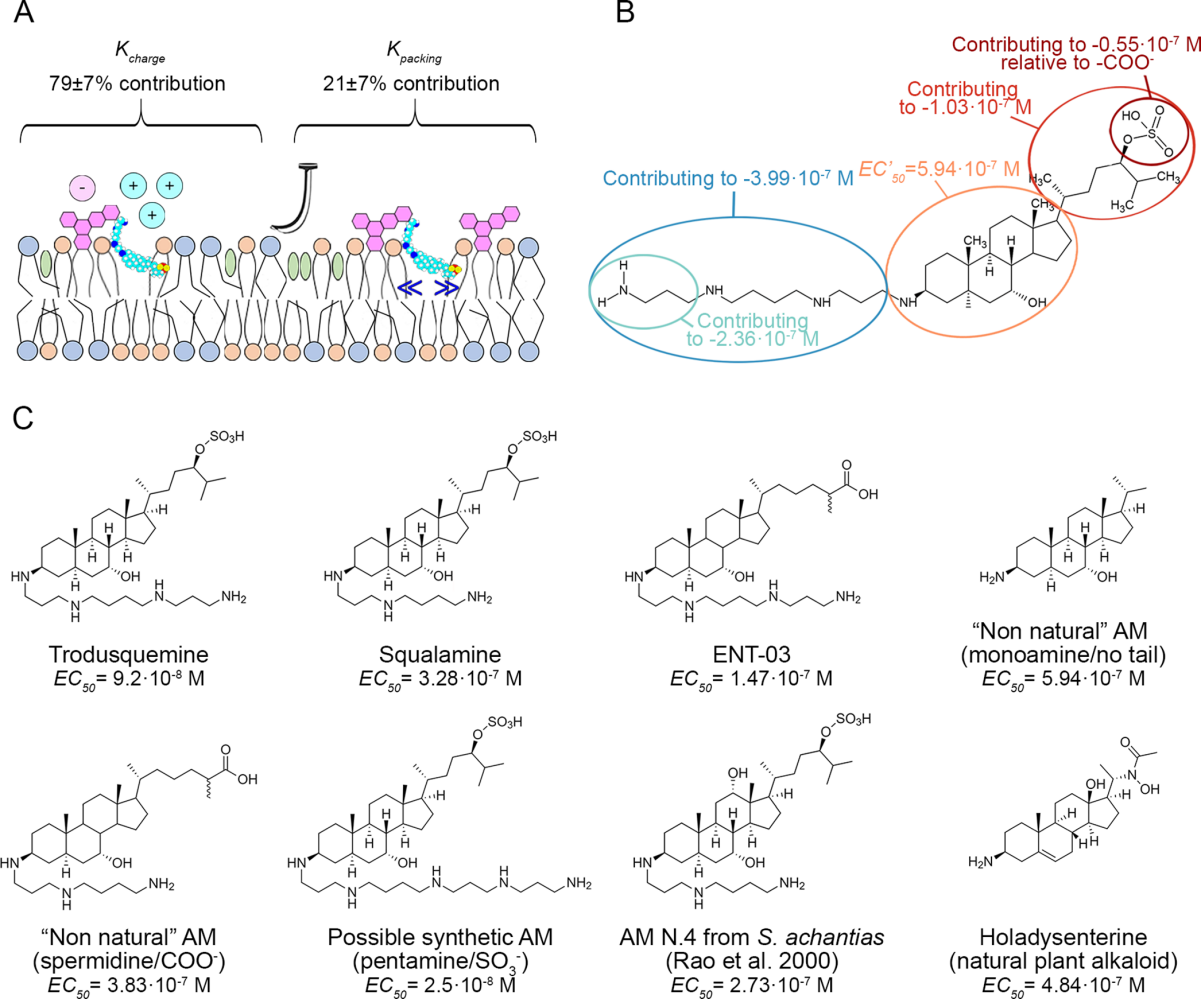
Contributions of the various membrane alterations and chemical groups of AMs to their potency in this experimental setting. (A) Contributions of the various types of membrane alterations and (B) of the chemical groups of AMs to the EC_50_ parameter in this experimental setting. The numbers reported in panel B refer to the contributions to the AM potency in membrane protection of SH-SY5Y cultured cells against misfolded protein oligomers of Aβ (ADDLs, 1 μm monomer equivalents) causing Ca^2+^ influx. The chemical formula in the image refers to TRO. (C) Representative AMs with their potencies (EC_50_ values) predicted in our experimental setting using the values reported in panel B. The EC_50_ values predicted for TRO, SQ, and ENT-03 are in good agreement with those observed experimentally.

Using a global fitting analysis, we described quantitatively the level of AM-mediated protection of the cell membrane as a function of all these factors, which allows the quantification of the weights that the different types of membrane alterations have in this protection (eq 4) and the contributions of the various chemical groups of AMs in their protective mechanism against oligomers (eq 5). In particular, the results of the global fitting analysis presented in the previous two sections allow us to calculate the contributions of the various types of membrane alterations (Figure 10A) and chemical groups of AMs (Figure 10B) to the EC_50_ parameter in the experimental setting described here based on cultured SH-SY5Y cells and Ca^2+^ influx measurements as a readout of membrane destabilization. They also provide hints to anticipate the effects, in a similar experimental setting, of other AMs isolated from sharks,^18^ AMs present in other animals that will probably be discovered in the next few years, monoamino-steroid molecules present in plants,^34−36^ and synthetic AMs (Figure 10C). Hence, these results help establish molecular principles for the further study and rational optimization of aminosterols and, more generally, help elucidate the means by which the physico-chemical properties of cell membranes can be targeted pharmacologically.

## Experimental Section

### Preparation of Large Unilamellar Vesicles (LUVs)

Liposomes were produced with a lipid mixture composed of 1,2-dioleoyl-sn-glycero-3-phosphocoline (DOPC, Avanti Polar Lipids) and sphingomyelin (SM, Sigma-Aldrich) in a molar ratio of 2:1 (mol/mol), 1% (mol) cholesterol (CHOL, Sigma-Aldrich), and 1% (mol) monosialotetrahexosylganglioside 1 (GM1, Avanti Polar Lipids). The lipids were dissolved in chloroform/methanol (2:1), and the organic solvent was removed by evaporation *in vacuo* (Univapo 150H, UniEquip) for 180 min. The mixture was hydrated at a total lipid concentration of 1.0 mg/mL with distilled water to form multilamellar vesicles (MLVs), left to swell for 1 h at 60 °C, and then extruded 17 times through a polycarbonate membrane with 100 nm pores using a miniextruder (Avanti Polar Lipids) at the same temperature, to form large unilamellar vesicles (LUVs). After cooling to room temperature, LUVs were stored at 4 °C for a maximum of 1 week. For the measurement of the ζ potential and BTF, and for the lipid-lipid FRET experiments, 5 μM of each AM was added during the hydration phase of LUVs preparation.

### Labeling of Aminosterols with BODIPY TMR and Alexa Fluor 594

SQ and TRO were synthetized by coupling spermidine and spermine, respectively, to the (5α,7α,24*R*)-3-keto-7-hydroxycholestan-24-ol sulfate steroid intermediate as previously described.^37−39^ The synthesis of ENT-03 was carried out similarly to the other AMs, by coupling a polyamine tail to a steroidal skeleton;^16^ the step by step procedure is reported in a deposited patent and will be published in a separate paper (Patent CN114929724; 2022). The >95% chemical purities of all AMs were assessed by HPLC-ELSD (Figures S5–S7) and ^1^H-NMR (Figures S8–S10). All AMs were stored as powders until use. For the labeling procedure, AMs were dissolved in distilled water to obtain a 100 mM stock solution and stored at 4 °C. BODIPY TMR-X NHS Ester and Alexa Fluor 594 NHS Ester (BODIPY and A594, respectively, ThermoFisher Scientific) were both dissolved in anhydrous DMSO to obtain 15 and 10 mM stock solutions, respectively, and stored at −20 °C. For labeling, 5 mM AM, 0.5 mM dye, 0.1 M sodium bicarbonate buffer, pH 8.3 for BODIPY and pH 7.0 for A594, were incubated in a final volume of 20 μL at 25 °C for 2 h under mild orbital shaking. During labeling with BODIPY the AM precipitates, therefore, after the incubation, the solution was centrifuged at 18,000*g* for 15 min; the pellet was dried with a nitrogen flow and resuspended in 20 μL DMSO to maintain the initial concentrations. During labeling with A594, TRO remains in solution, whereas SQ and ENT-03 precipitate. Hence, the solution with TRO labeled with A594 was directly used after incubation, while those with SQ and ENT-03 were centrifuged and resuspended in DMSO as described for the BODIPY labeling. With these procedures, the labeled:total AM was 1:10 in all cases. No unreacted dye was detected using mass spectrometry, following a previously described procedure.^11^ As a negative control, L-Arg was labeled with both BODIPY and A594 under the same conditions used for AM labeling and no precipitate was observed.

### Fluorescence Anisotropy of Fluorescently Labeled Aminosterols

BODIPY or A594-labeled AMs and L-Arg (negative control) were diluted with distilled water to 10 μM. The fluorescence anisotropy (*r*) values were then acquired at 570 nm after excitation at 544 nm and at 617 nm after excitation at 590 nm, respectively, in the absence and presence of 0.5 mg/mL unlabeled LUVs incubated for 15 min in the dark, using a 3 × 3 mm black walls quartz cell at 25 °C on an Agilent Cary Eclipse spectrofluorometer (Agilent Technologies) equipped with a thermostatted cell holder attached to an Agilent PCB 1500 water Peltier system.

### Fluorescence Emission of Fluorescently Labeled Aminosterols

BODIPY or A594-labeled AMs and L-Arg (negative control) were diluted with distilled water to 10 μM. Fluorescence emission spectra of AMs and L-Arg labeled with BODIPY and A594 were acquired from 550 to 650 nm (excitation at 544 nm) and from 600 to 700 nm (excitation at 590 nm), respectively, in the absence and presence of 0.5 mg/mL unlabeled LUVs incubated for 15 min in the dark, using the cell and spectrofluorometer described above.

### Binding Assay of Fluorescently Labeled Aminosterols and LUVs

BODIPY or A594-labeled AMs and L-Arg (negative control) were diluted with distilled water to 10 μM and incubated with increasing concentrations of unlabeled LUVs (from 0.0 to 1.0 mg/mL) for 15 min at 25 °C in the dark. Fluorescence emission of BODIPY and A594-labeled AMs and L-Arg were then acquired at 572 nm (excitation at 535 nm), and at 612 nm (excitation at 590 nm), respectively, using the cell and spectrofluorometer described above. The weak fluorescence contribution of unlabeled LUVs was subtracted from fluorescence emission spectra, and resulting values were then normalized to the value obtained in the absence of LUVs (taken as 100%). The fluorescence emission intensity was then plotted versus LUV concentration, and data points were then fitted with:
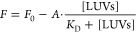
6where *F* is the fluorescence intensity at a given LUV concentration, *F*_0_ is fluorescence intensity at 0.0 mg/mL LUVs, *A* is the difference between the fluorescence emission of unbound and bound AMs, and *K*_D_ is the dissociation constant of the LUV-AM complex.

### Stopped-Flow Kinetic Analysis of TRO-LUV Binding

TRO-A594 (50 μM) was diluted 5-fold into solutions containing different concentrations of LUVs dissolved in H_2_O. We used a Bio-logic SFM-3 stopped-flow device (Claix, France) attached to a fluorescence detection system, an FC-08 cuvette (path length 0.08 cm), an excitation at 380 nm, and a band pass filter to collect emission above 475 nm. The flow rate was 2.19 mL/s. The injection time, total volume, and dead-time were 160 ms, 350 μL, and 14 ms, respectively. The final conditions after dilution were 10 μM TRO-A594, with LUV concentrations ranging from 0.12 to 1.00 mg/mL, 25 °C. Each trace was averaged over 2–7 experiments, normalized to the maximum fluorescence, and then analyzed with a double exponential equation:
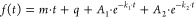
7where *f*(*t*) is the fluorescence recorded at time *t*, *m* and *q* are the slope and intercept of the plateau signal, respectively, *A*_1_ and *A*_2_ the amplitudes of the exponential phases, and *k*_1_ and *k*_2_ are their apparent rate constants. Plots of *k*_1_ and *k*_2_ versus LUV concentration were fitted to straight lines:^40^
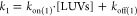
8
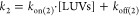
9

### Light Scattering Analysis of LUVs in in the Presence of Saturating Concentrations of Aminosterols

LUVs were diluted with distilled water to 0.5 mg/mL and incubated for 15 min at 25 °C with increasing concentrations of AMs (from 0 to 100 μM). The size distributions (light scattering versus apparent hydrodynamic diameter) and count rate (kilocounts per second, kcps) were then recorded at 25 °C, using a Zetasizer Nano S or APS (Malvern), thermostatted with a Peltier temperature controller, measurement position 4.20 mm, attenuator 6, and using disposable low volume (45 μL) plastic cuvettes. According to the laws of light scattering, the following equation holds:
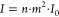
10where *I* is the total intensity of light scattered by LUVs in kcps, *n* is the number of LUVs, *m* is the mass of a single LUV, and *I*_0_ is the intensity of light scattered by a single unitary mass of LUV in kcps. LUV hydrodynamic diameter did not change with addition of any of the AMs, indicating that *n* and *I*_0_ remain constant. By contrast, when the AM is incorporated into the LUVs, *m* increases. The relative increase of LUV mass determined by the addition of AMs was then calculated with:
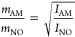
11where *I*_AM_ and *I*_NO_ are the intensities of light scattered by LUVs in the presence and absence of a given concentration of AM and *m*_AM_ and *m*_NO_ are the LUV mass concentrations in the presence and absence of a given concentration of AM. The obtained data were then plotted versus AM concentration.

### Microfluidics of TRO in the Presence of LUVs

LUVs were diluted with distilled water to 0.5 mg/mL and incubated for 15 min at 25 °C with 10–100 μM TRO-A594 or 20 μM TRO-BODIPY (1:10 dye:TRO) or 2 μM CHOL-BODIPY (1:1 dye:CHOL); samples with 10 or 50 μM TRO-A594 without LUVs were also prepared. The diffusion of the fluorescently labeled molecule in the various samples was evaluated with the microfluidic technique using a Fluidity One-W instrument (Fluidic Analytics) and placing a 5 μL drop of the sample on a disposable microfluidic chip made of cyclic olefin copolymer (COC) manufactured using injection molding (Fluidic Analytics). The diffusion was evaluated as the ratio of fluorescence values in the diffused versus that in the undiffused channels (*F*_d_/*F*_und_) or as the ratio of fluorescence values in the diffused channel versus total fluorescence [*F*_d_/(*F*_d_ + *F*_und_)]. This ratio parameter correlated directly with diffusion rapidity and inversely with size of the fluorescent molecule or its complex with LUVs. When this value was in the appropriate range, it was converted automatically by the instrument into a hydrodynamic radius (*R*_h_).

### ζ Potential Measurements

Zeta potential (ζ) measurements were performed with a Zetasizer Pro Red Label (Malvern). LUVs were covesiculated with 5 μM of each AM at a total lipid concentration of 1 mg/mL. About 600 μL of each LUV sample was diluted to obtain a total lipid concentration of 0.25 mg/mL, with phosphate buffer, 5.57 mM ionic strength, pH 7, 20 °C, and put in a disposable folded capillary cell (polycarbonate, Malvern). Each ζ potential value is the average of three independent runs; for each temperature, the ζ potential was determined as the mean of 5–8 measurements. The reported error is the standard deviation of the measurements. The measurements were performed in the range 10–60 °C, every 2 °C (except every 1 °C in the range 38–50 °C for ENT-03 containing LUVs, to better appreciate the transition). The electrophoretic mobility measurements were converted into ζ values according to the Smoluchowsky model.^41^ The temperature was internally controlled (accuracy ±0.1 °C). The ζ potential measurements were also used to determine the transition temperature (*T*_m_) in LUV systems.^42^ The *T*_m_ values were determined by analyzing the first-order derivative of ζ with respect to temperature (dζ/d*T*) as a function of temperature: the *T*_m_ corresponds to the minimum of the curve, while the amplitude of the transition was assumed to correspond to the full width at half maximum (FWHM) of the derivative curves.

### Atomic Force Microscopy (AFM)

LUVs were prepared at a total lipid concentration of 1.0 mg/mL covesiculated with 5 μM of each AM. Supported lipid bilayers (SLBs) were obtained by depositing 40 μL of each LUV suspension (after a ten-fold dilution) and 10 μL of a 10 mM CaCl_2_ solution onto a 1.0 × 1.0 cm^2^ freshly cleaved mica substrate. The samples were stored for 10 min at room temperature and then incubated for 15 min at 60 °C in a closed chamber at 100% relative humidity. The samples were cooled down at room temperature for 2 h and finally gently rinsed with Milli-Q water to remove nondeposited vesicles. Prior to AFM imaging, samples were kept again at room temperature in a closed chamber at 100% relative humidity. AFM imaging usually started 1.5 h after rinsing.

Force spectroscopy measurements were performed under a liquid environment with a Multimode SPM (Bruker) equipped with “E” scanning head (maximum scan size 15 μm) and driven by a Nanoscope V controller (Bruker). Triangular silicon nitride cantilevers (DNP-10, Bruker, nominal spring constant 0.24 N/m) were used. The actual spring constant of each cantilever was determined *in situ* using the thermal noise method.^43^ Force maps consisting of 128 × 128 force distance curves were acquired point-by-point on scan areas of 5 × 5 μm^2^ or 2.5 × 2.5 μm^2^. The maximum force load was 15–18 nN. Breakthrough forces were evaluated from the force–distance curves data sets using a home-built software.

### Lipid–Lipid FRET

LUVs were prepared at a total lipid concentration of 1.0 mg/mL, as described above. TRO, SQ, and ENT-03, when present, were added during the hydration phase to a final concentration of 5 μM. BODIPY-FL C5-ganglioside GM1 (GM1-D), BODIPY-FL-cholesterol (CHOL-D), BODIPY-FL-sphingomyelin (SM-D, commercial name TopFluor Sphingomyelin, Avanti Polar Lipids), and BODIPY-FL-DOPC (DOPC-D, commercial name TopFluor PC, Avanti Polar Lipids) were used as donor lipids. Cholesteryl 4,4-difluoro-5-(4-methoxyphenyl)-4-bora-3a,4a-diaza-s-indacene-3-undecanoate (CHOL-A, commercial name CholEsteryl BODIPY 542/563 C11, ThermoFisher Scientific) was used as an acceptor lipid. The molar fraction of each lipid labeled with D or with A was 0.0625% of total lipids in all cases.

Fluorescence spectra of LUVs containing only lipid-D, only CHOL-A, and both lipid-D and CHOL-A were acquired using the cell and spectrofluorometer described above, at 25 °C, with excitation at 450 nm and emission from 480 to 640 nm. FRET efficiencies (*E*) were calculated as
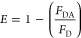
12where *F*_DA_ is the fluorescence intensity of D in the presence of A, and *F*_D_ is the fluorescence intensity of D in the absence of A.^44^ FRET *E* calculated with eq 12 was converted into distance between D and A (*r*) using:^44^
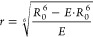
13where *R*_0_ is the Forster distance and was previously calculated for this D/A probe pair.^11^

### FAR-UV CD Spectroscopy

αS (5 μM) was incubated with DMPS LUVs (250 μM total lipids, corresponding to 0.2 mg/mL total lipids) for 30 min and then with increasing concentrations of TRO, SQ, or ENT-03 (0, 10, 20, 30, 40, 50, 60, and 70 μM) for 15 additional min, in 20 mM sodium phosphate buffer, pH 6.5, at 30 °C. LUVs were prepared with the same procedure reported above, but using 100% DMPS. Far-UV CD spectra were recorded on a Jasco J-810 spectropolarimeter equipped with a thermostated cell holder attached to a Thermo Haake C25P water bath using a quartz cuvette with path length of 1 mm, at 30 °C. CD spectra were recorded from 180 to 260 nm by averaging 5 spectra with a data pitch of 0.2 nm, a scanning speed of 50 nm/min, and a response time of 1 s. All spectra were blank subtracted and truncated at HT > 700 V, then normalized to mean molar residue ellipticity using:
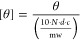
14where [θ] is the mean residue ellipticity in deg cm^2^ dmol^–1^, θ is the ellipticity in mdeg, *N* is the number of residues, *d* is the optical path in cm, *c* is the concentration in g/l, and mw is the molecular weight in g/mol. For all analyses, [θ] at 222 and 192 nm was plotted as a function of AM concentration.

### Preparation of Aβ_42_ ADDLs

Lyophilized Aβ_42_ (Bachem) was dissolved in HFIP to 1.0 mM and incubated for 1 h at room temperature to allow complete peptide monomerization. Aβ_42_-derived diffusible ligands (ADDLs) were prepared as described previously.^45^ In particular, the HFIP was evaporated with a gentle flow of N_2_ and the dried protein was resuspended to 5 mM with DMSO and then diluted with phenol red free F-12 HAM to 100 μM. The sample was then incubated at 4 °C for 24 h and centrifuged at 12000*g* for 10 min, 4 °C, to collect the supernatant containing the oligomers.

### Cell Culture

Authenticated human SH-SY5Y neuroblastoma cells were purchased from A.T.C.C. and cultured in Dulbecco’s Modified Eagle’s Medium (DMEM), F-12 Ham with 25 mM 4-(2-hydroxyethyl) piperazine-1-ethanesulfonic acid (HEPES), and NaHCO_3_ (1:1) supplemented with 10% fetal bovine serum (FBS), 1 mM glutamine, and 1% penicillin and streptomycin solution (Sigma-Aldrich). Cells were maintained in a 5% CO_2_ humidified atmosphere at 37 ° C, grown until 80% confluence for a maximum of 20 passages, and routinely tested to ensure that they were free form mycoplasma contamination.^46^ The cell line was authenticated by the European Collection of Authenticated Cell Cultures using short tandem repeat loci analyses.

### Binding of Aminosterols to Cells

SH-SY5Y cells were plated in 12-well plates containing coverslips at a density of 50,000 cells per well. 24 h after plating, the cells were washed with phosphate-buffered saline (PBS) and incubated at room temperature for 30 min with 5 μM TRO-BODIPY, SQ-BODIPY, ENT-03-BODIPY, or L-Arg-BODIPY (1:10 dye:molecule) diluted in the Leibovitz’s L-15 (ThermoFisher Scientific), a medium designed for supporting cell growth in the absence of CO_2_ equilibration. Ten min before the incubation ending, the Hoechst 33342 dye was added to the culture medium (10 μg/mL). The analysis of AM-derived fluorescence and nuclei-derived fluorescence were performed on a Nikon Eclipse TE300 C2 confocal laser scanning microscope (Nikon) equipped with a Nikon 60x immersion oil objective (Apo Plan, NA 1.4) and with Coherent CUBE (diode 405 nm) and Coherent Sapphire (Sapphire 561 nm) lasers. The emission filters for imaging were 452/45 and 595/60 nm. All settings, including pinhole diameter, detector gain and laser power, were optimized for each analysis.

### Measurement of Cytosolic Ca^2+^ Levels

SH-SY5Y cells were plated in 12-well plates containing coverslips at a density of 40,000 cells per well. 24 h after plating, the cells were washed with PBS and incubated at 37 °C for 15 min with ADDLs (1 μM, monomer equivalents) in the absence or presence of increasing concentrations (0.05, 0.1, 0.5, 1, 2.5, and 5 μM) of TRO, SQ, or ENT-03. Cytosolic Ca^2+^ levels were measured in living SH-SY5Y cells after the different treatments, by loading the cells with 4 μM Fluo-4 AM (Thermo Fisher Scientific) for 10 min, as previously reported.^46^ Ca^2+^ levels were detected after excitation at 488 nm and emission at 520–580 nm, by a TCS SP8 scanning confocal microscopy system (Leica Microsystems), equipped with an argon laser source. A series of 1 μm thick optical sections (1024 × 1024 pixels) were taken through the cell depth for each sample using a Leica Plan Apo 63× oil immersion objective, and all sections were projected as a single composite image by superimposition. The confocal microscope was set at optimal acquisition conditions, e.g., pinhole diameters, detector gain, and laser powers. Settings were maintained constant for each analysis. Images were then analyzed using the ImageJ (NIH) software (Rasband 1997–2018). Fluorescence intensities were typically expressed as a percentage of that measured in untreated cells.

## Supporting Information Available

Representative trace of stopped flow experiments, showing the fluorescence emitted during the binding of 10 μM TRO-A594 with 0.20 mg/mL LUVs (Figure S1); DLS size distributions of LUVs incubated with the three AMs (Figure S2); microfluidics of different concentrations of TRO-A594 in the absence or presence of LUVs (Figure S3); plot reporting theoretical versus experimental *R* values obtained using the leave-one-out cross-validation (LOOCV) method (Figure S4); chemical purity assessed by HPLC-ELSD of SQ, TRO, and ENT-03 (Figures S5–S7); chemical identity assessed by ^1^H-NMR of SQ, TRO, and ENT-03 (Figure S8–10); and chemical structures and molecular formula strings of SQ, TRO, and ENT-03 (Figure S11) (PDF)
TRO, SQ, and ENT-03 structures and corresponding formula strings (CSV)

## Abbreviations Used

A: acceptor
A594: Alexa Fluor 594 NHS Ester
AFM: atomic force microscopy
AM-A594: Alexa Fluor 594-labeled AM
AM-BODIPY: BODIPY TMR-X-labeled AM
AMs: aminosterols
BODIPY: BODIPY TMR-X NHS Ester
BTF: breakthrough force
CHOL: cholesterol
CHOL-A: Cholesteryl 4,4-difluoro-5-(4-methoxyphenyl)-4-bora-3a,4a-diaza-s-indacene-3-undecanoate
CHOL-D: BODIPY-FL-cholesterol
COC: cyclic olefin copolymer
D: donor
DMEM: Dulbecco’s Modified Eagle’s Medium
DOPC: 1,2-dioleoyl-sn-glycero-3-phosphocoline
DOPC-D: BODIPY-FL-DOPC
FBS: fetal bovine serum
F: fluorescence
FTD: frontotemporal dementia
GM1: monosialotetrahexosylganglioside 1
GM1-D: BODIPY-FL C5-ganglioside GM1
HEPES: 4-(2-hydroxyethyl) piperazine-1-ethanesulfonic acid
LOOCV: leave-one-out cross-validation
LUVs: large unilamellar vesicles
MLVs: multilamellar vesicles
PrP^sc^: prion protein
R: normalized response
*R_h_*: hydrodynamic radius
SE: spongiform encephalopathies
SLBs: supported lipid bilayers
SM-D: BODIPY-FL-sphingomyelin
SQ: squalamine
SUVs: small unilamellar vesicles
*T*_m_: transition temperature
TRO: trodusquemine
αS: α-synuclein
ζ: zeta potential
