# Supplementary material for: Quantitative Attribution of the Protective Effects of Aminosterols against Protein Aggregates to Their Chemical Structures and Ability to Modulate Biological Membranes
Source: J Med Chem. 2023 Jul 11;66(14):9519–36. doi: 10.1021/acs.jmedchem.3c00182 (PMC10388293; doi:10.1021/acs.jmedchem.3c00182)
Supplement: Supplementary file 1 — jm3c00182_si_001.pdf [file jm3c00182_si_001.pdf]

Supporting Information

Quantitative Attribution of the Protective Effects of Aminosterols Against Protein Aggregates to their Chemical Structures and Ability to Modulate Biological Membranes

Silvia Errico,[a,b] Giacomo Lucchesi,[c] Davide Odino,[d] Enass Youssef Osman,[a,e] Roberta Cascella,[a] Lorenzo Neri,[a] Claudia Capitini,[f,g,h] Martino Calamai,[f,h] Francesco Bemporad,[a] Cristina Cecchi,[a] William A. Kinney,[i] Denise Barbut,[i] Annalisa Relini,[d] Claudio Canale,[d] Gabriella Caminati,[c] Ryan Limbocker,[j] Michele Vendruscolo,[b] Michael Zasloff,[i,k] Fabrizio Chiti[a,*]

[a]*Dept of Experimental and Clinical Biomedical Sciences, Section of Biochemistry, University of Florence, Florence 50134, Italy*
[b]*Centre for Misfolding Diseases, Department of Chemistry, University of Cambridge, Cambridge CB2 1EW, UK*
[c]*Dept of Chemistry "Ugo Schiff" and CSGI, University of Florence, Sesto Fiorentino 50019, Italy*
[d]*Dept of Physics, University of Genoa, Genoa 16146, Italy*
[e]*Dept of Pharmacology and Toxicology, Faculty of Pharmacy, Tanta university, Tanta 31527, The Arab Republic of Egypt*
[f]*European Laboratory for Non-linear Spectroscopy (LENS), Sesto Fiorentino 50019, Italy*
[g]*Dept of Physics and Astronomy, University of Florence, Sesto Fiorentino 50019, Italy*
[h]*National Institute of Optics, National Research Council of Italy (CNR), Florence 50125, Italy*
[i]*Enterin Research Institute Inc., Philadelphia 19103, Pennsylvania, USA*
[j]*Dept of Chemistry and Life Science, United States Military Academy, West Point 10996, New York, USA*
[k]*MedStar-Georgetown Transplant Institute, Georgetown University School of Medicine, Washington DC 20007, USA*

* To whom correspondence should be addressed. E-mail: fabrizio.chiti@unifi.it

Table of content Supporting Information

Representative trace of stopped flow experiments, showing the fluorescence emitted during the binding of 10 μM TRO-A594 with 0.20 mg/ml LUVs (**Fig. S1**); DLS size distributions of LUVs incubated with the 3 AMs (**Fig. S2**); Microfluidics of different concentrations of TRO-A594 in the absence or presence of LUVs (**Fig. S3**); Plot reporting theoretical versus experimental R values obtained using the leave-one-out cross-validation (LOOCV) method (**Fig. S4**); Chemical purity assessed by HPLC-ELSD of SQ, TRO and ENT-03 (**Fig. S5-S7**); Chemical identity assessed by ^{1}H-NMR of SQ, TRO and ENT-03 (**Fig. S8-10**); Chemical structures and molecular formula strings of SQ, TRO and ENT-03 (**Fig. S11**).

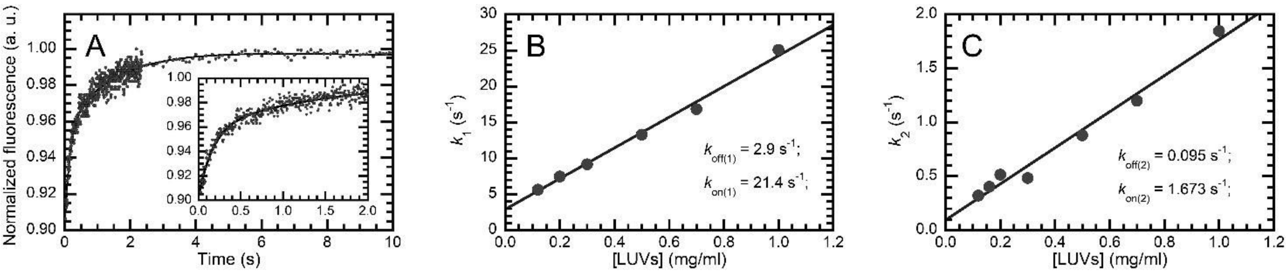

Figure S1. **Investigation of the binding between TRO-A594 and LUVs in real-time. (A)** Representative trace, showing the fluorescence emitted during the binding of 10 μM TRO-A594 with 0.20 mg/ml LUVs. The inset shows the first two seconds of recording, to highlight the first fast phase. The continuous line represents the best fit of experimental data to Eq. 7. **(B,C)** Plots of the apparent rate constants k_1 **(B)** and k_2 **(C)** obtained from best fits of the experimental traces to Eq. 7 *versus* [LUVs]. The continuous lines represent linear fits of the experimental values to Eq. 8 **(B)** and 9 **(C)**.

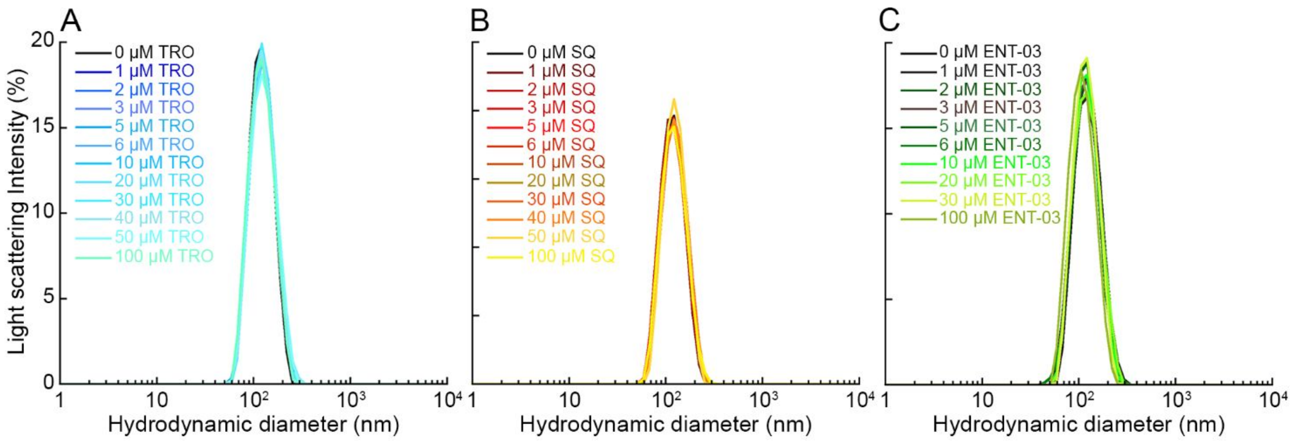

Figure S2. Size distributions of LUVs incubated with the 3 AMs. Plots reporting the size distributions of LUVs incubated with the indicated increasing concentrations of TRO **(A)**, SQ **(B)** and ENT-03 **(C)**.

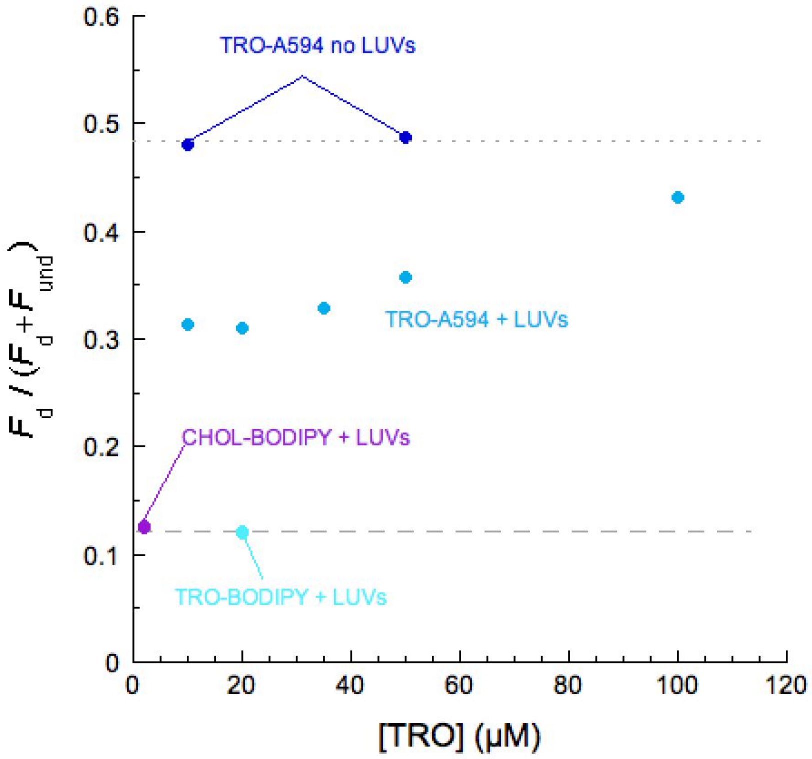

Figure S3. Microfluidics of different concentrations of TRO-A594 in the absence or presence of LUVs. Ratio of fluorescence values in the diffused channel *versus* total fluorescence [$F_d/(F_d+F_{und})$], determined with the microfluidic technique, for 10 µM and 50 µM TRO-A594 (1:10 of dye:TRO) in the absence of LUVs (dark blue), for 10-100 µM TRO-A594 (1:10 of dye:TRO) in the presence of 0.5 mg/ml LUVs (n=1, medium blue), for 20 µM TRO-BODIPY (1:10 of dye:TRO) in the presence of 0.5 mg/ml LUVs (pale blue) and for 2 µM CHOL-BODIPY (1:10 of dye:TRO) in the presence of 0.5 mg/ml LUVs used as a positive control of a molecule bound to LUVs (purple). The high value of $F_d/(F_d+F_{und})$ measured for TRO-A594 is typical of a small highly diffusible molecule and led to a value of apparent hydrodynamic radius of 725±35 pm, consistent with that of a molecule of the size of TRO-Alexa-594 in a monomeric form (expected value of 700-800 pm). The small value of $F_d/(F_d+F_{und})$ measured for TRO-BODIPY in the presence of LUVs rules out that the molecule was even in part monomeric or assembled into low-molecular weight micelles and rather indicates that the molecule was entirely bound to LUVs, in agreement with the binding plot showing that binding saturation was achieved at these concentrations of TRO-BODIPY and LUVs (**Fig. 3A**). The similar value of $F_d/(F_d+F_{und})$ measured for CHOL-BODIPY in the presence of LUVs, used here as a positive control of a molecule bound to LUVs, confirmed full binding of TRO-BODIPY to LUVs. Values of $F_d/(F_d+F_{und})$ measured at low concentrations of TRO-A594, in the presence of LUVs, were intermediate, indicating partial binding of the labelled molecule to LUVs, in agreement with the binding plots showing pre-saturation conditions at these concentrations of TRO-A594 and LUVs (**Fig. 3B**). As the concentration of TRO-A594 increases, the value of $F_d/(F_d+F_{und})$ also increases, with a value at 100 µM TRO-A594 close to that of TRO-A594 in the absence of LUVs. This indicates that LUVs are saturated with TRO-A594 under these conditions and the excessive TRO-A594 pool remains unbound. The TRO-A594 concentration at which $F_d/(F_d+F_{und})$ starts to increase is roughly 28-35 µM, in agreement with that determined with the light scattering analysis (**Fig. 4A**). Moreover, the $F_d/(F_d+F_{und})$ value measured at 100 µM TRO-A594 is consistent with concentrations of ca. 65 µM and ca. 35 µM unbound and bound in pre-saturating conditions, respectively, lending further support to a saturating value of *ca.* 35 µM determined with the light scattering analysis.

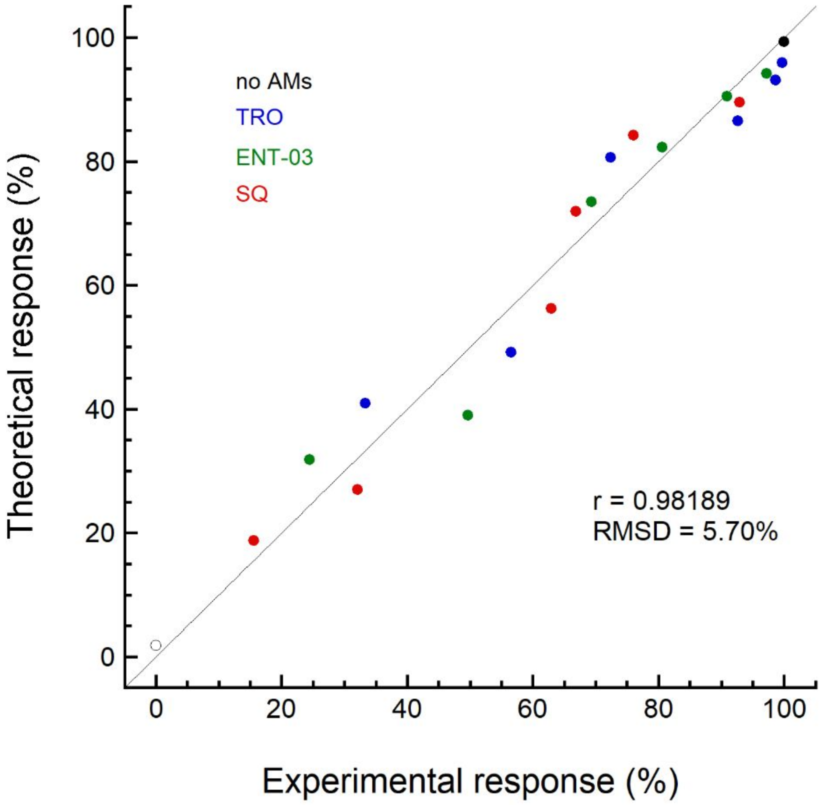

Figure S4. Plot reporting theoretical *versus* experimental *R* values obtained using the leave-one-out cross-validation (LOOCV) method. To obtain this plot, each experimental *R* value was left out from the analysis and Eq. 4 was re-determined using the remaining experimental *R* values (four constants and theoretical *R* value corresponding to the one left out).

**SQ Chemical Purity**						
Peak	Name	Retention Time (min)	RT ratio	Height	Area	Peak Area Percentage
1	Spermidin	1.543				
2	Azidospermidin	1.672				
3	L-(+)-Lattate	3.508				
4		4.293	0.62	1552.2	13119.42182	0.11651
5		4.948	0.72	2197.2	16562.04339	0.14709
6		5.171	0.75	516.9	3801.08108	0.03376
7		5.636	0.82	971.9	6198.00359	0.05504
8		6.081	0.88	1167.0	4443.27042	0.03946
9	24S-Bisnol	6.196	0.90	14182.5	72041.01922	0.63979
10	Bisnol	6.873		767195.5	10798745.245	95.90208
11	Lactylamid	7.957	1.16	6231.9	58590.71353	0.52034
12	3-alpha-Isomer	8.878				
13		9.620	1.40	3763.3	24673.37124	0.21912
14		10.581	1.54	4910.7	38441.39548	0.34139
15		10.723	1.56	1537.6	13902.63858	0.12347
16		11.495	1.67	864.1	6850.74893	0.06084
17		12.042	1.75	2057.3	24589.53302	0.21838
18		12.364	1.80	3916.4	54944.79968	0.48796
19	VU	12.548	1.83	1596.2	10964.14046	0.09737
20		14.246	2.07	5723.7	65318.68652	0.58009
21		14.429	2.10	5295.3	46991.77686	0.41733
Sum					11260177.88882	100.00000

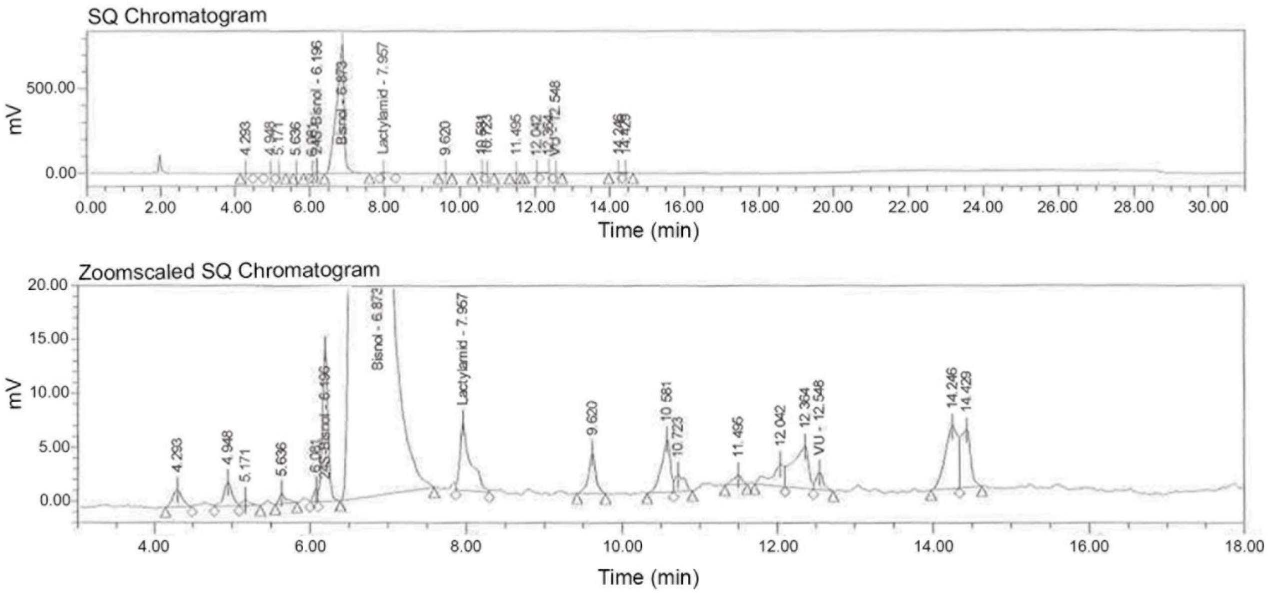

Fig. S5. Chemical purity (top) and HPLC chromatogram (bottom) of SQ

<table>
<tr><td colspan="5">TRO Chemical Purity</td></tr>
<tr><td colspan="2">Instrument</td><td colspan="3">Waters Acquity ELSD</td></tr>
<tr><td colspan="2">Mobile Phase</td><td colspan="3">A: 0.1% Formic Acid in Water
B: 0.1% Formic Acid in Acetonitrile</td></tr>
<tr><td colspan="2">Column</td><td colspan="3">Kinetex XB-C18, (2.1x75 mm, 1.7 µm)</td></tr>
<tr><td colspan="2">Gradient</td><td colspan="3">5-95%B over 8 min, hold 95% B 1 min</td></tr>
<tr><td colspan="2">Flow Rate</td><td colspan="3">0.6 ml/min</td></tr>
<tr><td colspan="2">Date</td><td colspan="3">12/10/2018</td></tr>
<tr><td>Peak</td><td>Retention Time (min)</td><td>Area (LSU)</td><td>Height (LSU)</td><td>Peak Area Percentage</td></tr>
<tr><td>1</td><td>2.10</td><td>65409.23</td><td>326265</td><td>95.13</td></tr>
<tr><td>2</td><td>2.78</td><td>3348.30</td><td>17071</td><td>4.87</td></tr>
</table>

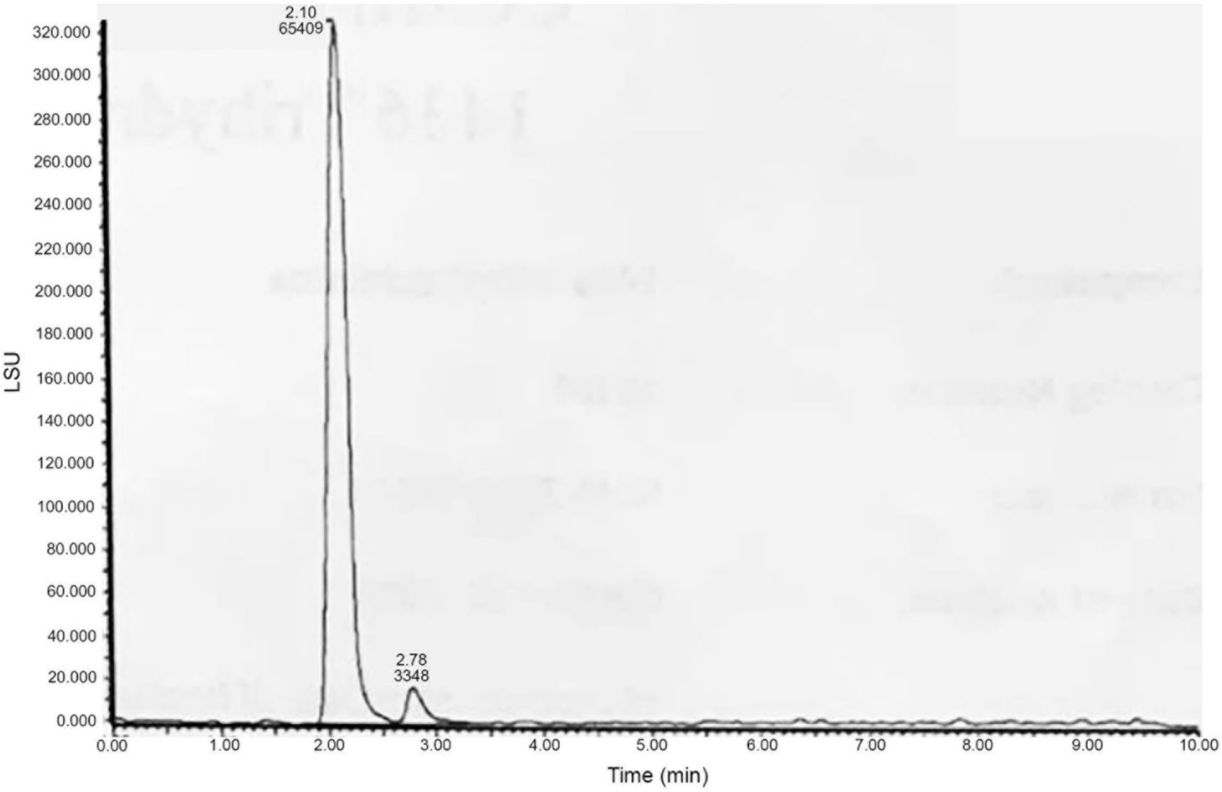

Fig. S6. Chemical purity (top) and HPLC chromatogram (bottom) of TRO

ENT-03 Chemical Purity				
Instrument			Agilent HPLC	
Detector			ELSD	
Mobile Phase			A: 0.1% Formic Acid in Water<br>B: 0.1% Formic Acid in Acetonitrile	
Column			Kinetex XB-C18, (2.1x75 mm, 1.7 µm)	
Gradient			5-95%/8 min, hold 95% B	
Flow Rate			0.6 ml/min	
Wavelength			ELSD	
Date			17/04/2020	
Results			95.0%	
Peak	Retention Time (min)	Area	Height	Peak Area Percentage
1	2.890	12.90338	0.713119	0.4721%
2	4.907	2597.03711	203.10667	95.0212%
3	5.551	116.51572	10.39299	4.2631%
4	7.149	6.65670	0.502358	0.2436%

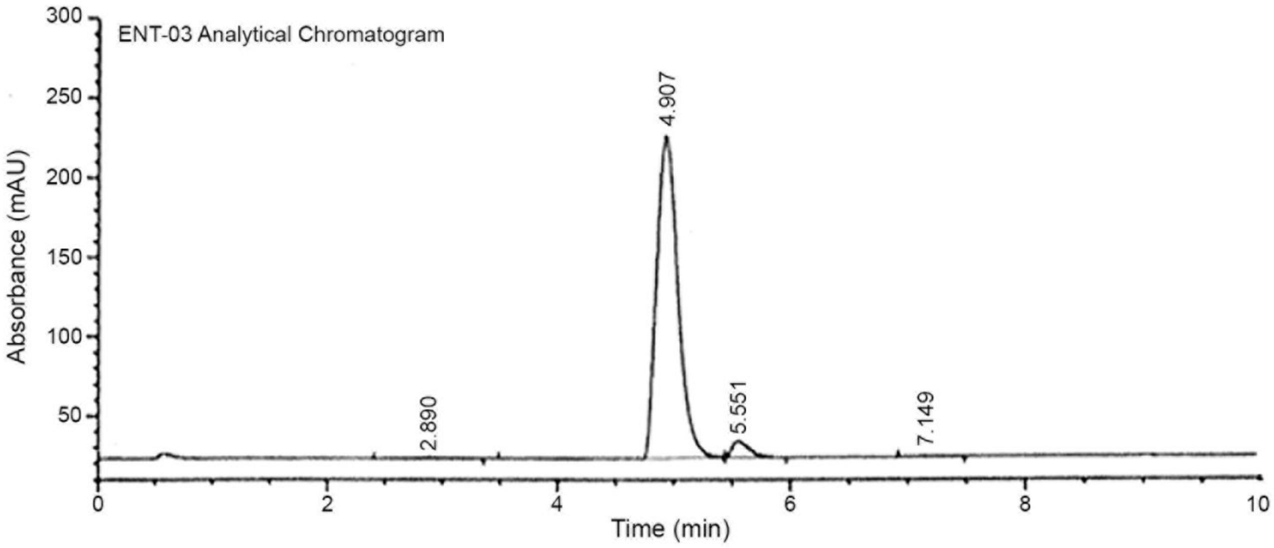

Fig. S7. Chemical purity (top) and HPLC chromatogram (bottom) of ENT-03.

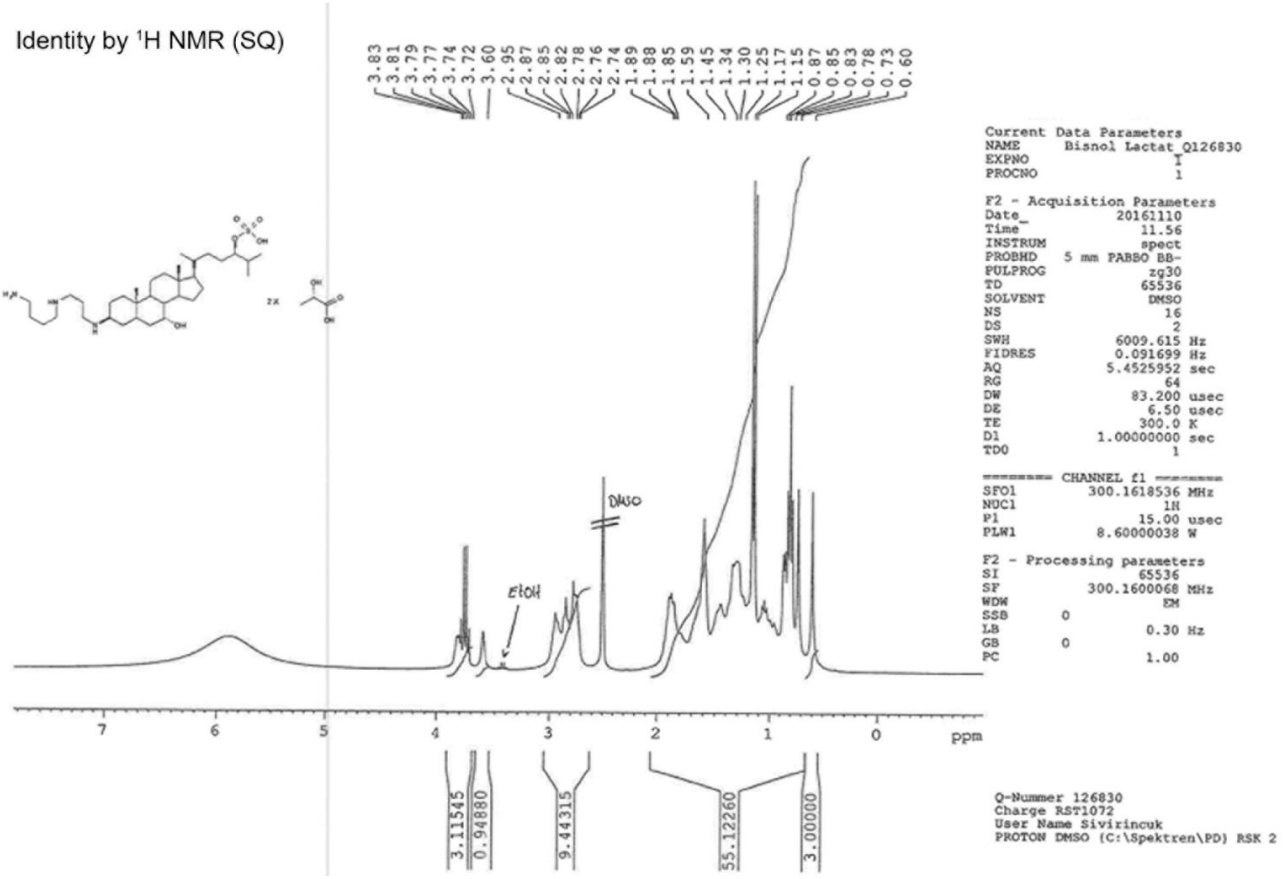

Fig. S8. ^{1}H-NMR spectrum of SQ

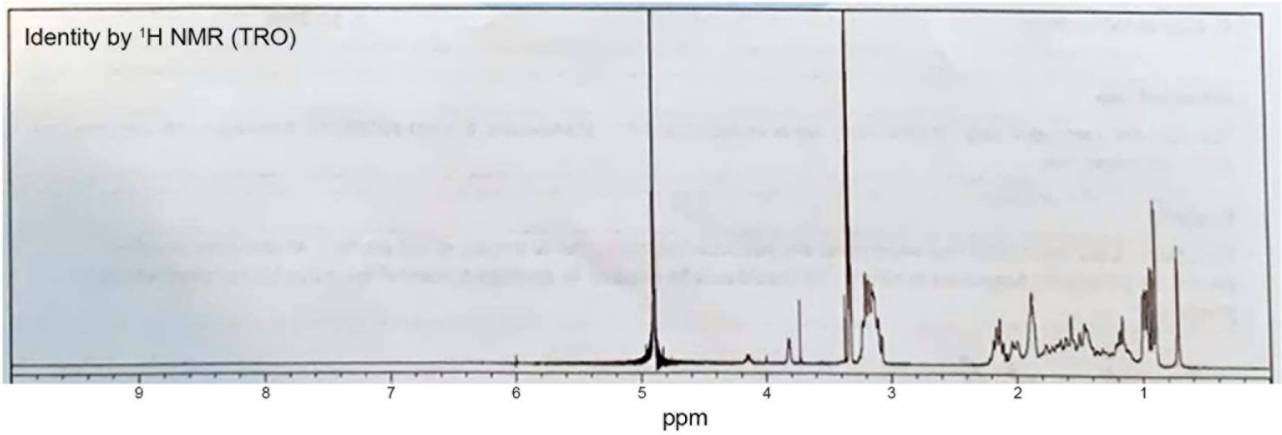

Fig. S9. ^{1}H-NMR spectrum of TRO

Identity by ¹H-NMR (ENT-03)	Instrument	Bruker 400MHz		
	Acquisition Date	3/26/2020	Solvent	D_2O

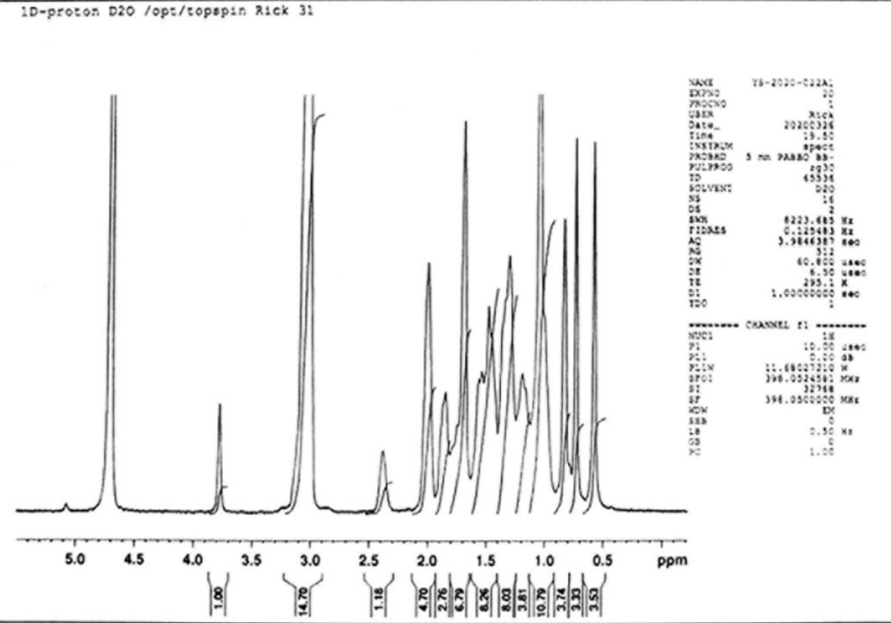

Fig. S10. ¹H-NMR spectrum of ENT-03

Compound	SMILES
Trodusquemine or MSI-1436 (TRO)	CC(C)[C@H](OS(=O)(=O)O)CC[C@@H](C)C1CCC2[C@H]3[C@H](CC[C@]12C)[C@@]1(C)CC[C@H](NCCCNCCCCNCCCN)C[C@@H]1C[C@H]3O
Squalamine (SQ)	CC(C)[C@H](OS(=O)(=O)O)CC[C@@H](C)C1CCC2[C@H]3[C@H](CC[C@]12C)[C@@]1(C)CC[C@H](NCCCNCCCCN)C[C@@H]1C[C@H]3O
ENT-03	O=C(O)C(C)CCC[C@@H](C)C1CCC2[C@H]3[C@H](CC[C@]12C)[C@@]1(C)CC[C@H](NCCCNCCCCNCCCN)C[C@@H]1C[C@H]3O

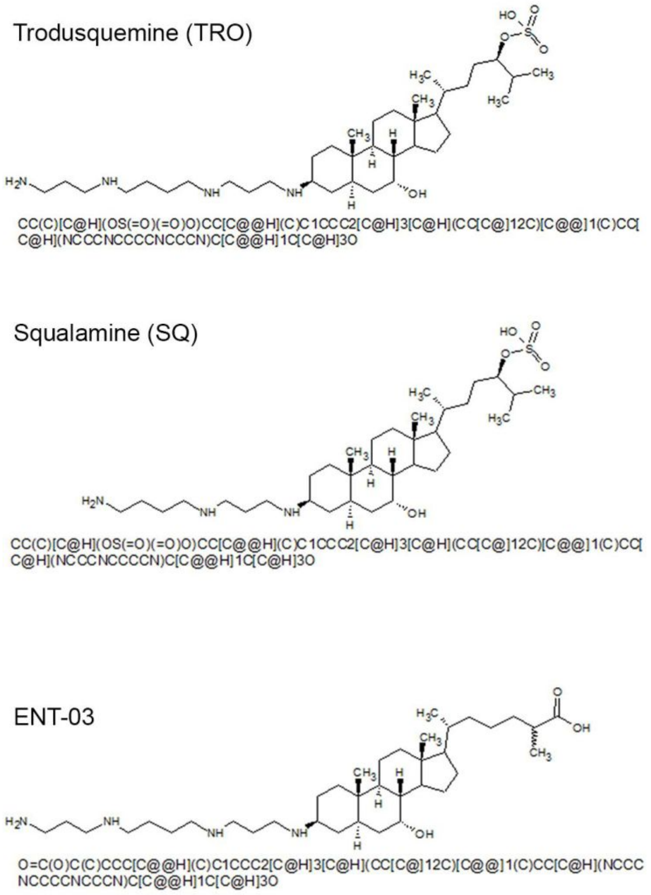

Fig. S11. TRO, SQ and ENT-03 structures and corresponding formula strings
